# Hypertuned boosting approach with Local Binary Pattern and Pivot Distribution Count method feature extractor for glaucoma identification

**DOI:** 10.1038/s41598-026-58711-8

**Published:** 2026-06-23

**Authors:** Santosh Kumar Majhi, Sushma Soni, Ankita Misra, Rosy Pradhan, Abhijeet Mahapatra

**Affiliations:** 1 Department of Computer Science and Information Technology, Guru Ghasidas Vishwavidyalaya (A Central University), Bilaspur, 495009 Chhattisgarh India; 2 Department of Computer Science and Engineering, Veer Surendra Sai University of Technology, Burla, 768018 India; 3 Department of Electrical Engineering, Veer Surendra Sai University of Technology, Burla, 768018 India; 4 Manipal Institute of Technology Bengaluru, Manipal Academy of Higher Education, Manipal, India

**Keywords:** Fundus Images, Glaucoma, Hyperparameter tuning, Local binary pattern, Pivot Distribution Count, Computational biology and bioinformatics, Diseases, Engineering, Mathematics and computing

## Abstract

Glaucoma, the second largest cause of irreversible blindness worldwide, causes significant damage to the optic nerve. Early diagnosis of glaucoma is crucial since without it, there will be continuous deterioration of vision. Manual detection of glaucoma based on fundus images is a tedious and potentially inaccurate process, making it important to develop computer-aided glaucoma detection systems. This paper introduces a hybrid approach to glaucoma detection that involves the use of Local Binary Pattern (LBP) and Pivot Distribution Count (PDC) feature extraction methods for the analysis of retinal fundus images. LBP is a technique that involves the extraction of texture-based features, whereas PDC is a feature extraction algorithm that involves the extraction of white-pixel intensity and fractal dimension from retinal fundus images. In this study, features were extracted and classified using different machine learning algorithms such as SVM, Decision Trees (DT), Random Forest (RF), KNN, Adaboost, Gradient Boosting, XGboost, Light Gradient Boosting Machine, and CatBoost. Moreover, grid search cross validation, randomized search cross validation, genetic algorithm, and Bayesian optimization were also applied to optimize the performance of LightGBM and Catboost classifiers. The proposed Hybrid LBP-PDC method provided maximum classification accuracy of 97.55% using the CatBoost and LightGBM classifiers. Moreover, we have developed a robust and efficient methodology for the automatic glaucoma screening using the retinal fundus image analysis.

## Introduction

The most common cause of complete blindness in the world is Glaucoma, which is associated with progressive injury caused to the optic nerve, leading to poor vision, which, if not treated during its early stages, could result in complete blindness. Glaucoma mainly targets the retinal nerve fibre layer and optic nerve head, hence causing structural and functional abnormalities in the human eye. Since glaucoma develops very gradually without any signs in its early stages, it is very important to diagnose it early to avoid complete blindness. This is because early detection and monitoring can greatly help in reducing the effect of glaucoma on the patients’ quality of life^1^.

One of the most commonly used methods for glaucoma detection in medicine is retinal fundus imaging, which creates clear images of critical parts of the retina including the optic disc, optic cup, retinal nerve fibres, and blood vessels. These retinal features are detected by clinicians during manual examination to determine glaucoma. However, manual diagnosis is an inefficient, subjective process that also relies on the expertise of the practitioner, thus creating problems in terms of delays and inconsistency. For this reason, research into automated, computer-assisted glaucoma detection has increased in recent years^2^.

Existing in most glaucoma detection methods, which are mostly single-feature-based extraction methods, optic disc segmentation is either outsourced or handled by black-box Deep Learning (DL) models. While many Machine Learning (ML) and DL-based studies report very promising results, they are also marred by issues such as poor feature representation, low robustness, not-so-great validation methods, and poor generalization across different data sets. Also, most studies present either texture- or intensity-based analyses, which, in isolation, may not capture the full picture of glaucoma’s pathologic features in fundus images. To address these issues, this study presents a hybrid glaucoma detection framework that combines Local Binary Pattern (LBP) and Pivot Distribution Count (PDC) feature extraction methods to address both texture and white-pixel intensity in retinal fundus images. Also, in this work, we use several boosting algorithms, including LightGBM and CatBoost. We tune hyperparameters using methods such as GridSearchCV, RandomizedSearchCV, GA, and Bayesian Optimization to enhance the models’ classification performance. Also, we use a 5-fold cross-validation procedure, for which we go through the processes of breaking our training data into 5 groups, which we then use in turn for testing while the other 4 groups form the train set this is done to better prepare the model, to also reduce the bias at the same time which in turn also will add to the quality of the reported experiment results. Also, in contrast to what is usually done, we present a framework that includes handmade texture analysis, intensity-based feature extraction, hyperparameter tuning, and robust validation strategies, which we propose to greatly improve performance in glaucoma classification. Also, our proposed method, in its design, aims at what is easy to compute and to also report which, in practice, is what we did: we made it not be a very complex black box system, as some DL models are, which also makes our model more applicable for a real-world computer-aided glaucoma screen, which was our main goal.

### Motivation

The rapid increase in glaucoma cases is a major issue in ophthalmology, which we see because the disease progressively affects the optic nerve and may lead to blindness, which is permanent if not treated. Also, we see that early symptoms of glaucoma are hard to identify, which in turn means many patients go undiagnosed until they have severe vision loss. We are to see the rise of fundus imaging as a valuable clinical tool for studying glaucoma-related retinal abnormalities, including changes in the optic disc and retinal nerve Fiber layer. But manual review of retinal fundus images is not only time-intensive but also largely at the expert ophthalmologist’s discretion, which, in turn, has made research into automatic glaucoma screening very important^1^.

Several ML and DL methods for automated glaucoma identification using retinal fundus pictures have been introduced in recent years. While these methods have reported very good results, most approaches at present use either texture- or intensity-based analysis, which does not, in fact, capture all the pathologic changes associated with glaucoma. Also, DL models require large-scale training datasets and substantial computational resources, which, in turn, limit their practical use in resource-constrained health care settings. Thus, what is needed is a more efficient and reliable system capable of extracting multiple key features from fundus images. Motivated by this, we present a hybrid glaucoma detection method that combines LBP and PDC feature extraction methods to analyze our retinal textural data and the white-pixel intensity distribution, thereby improving glaucoma classification performance^1,2^.

Many current glaucoma detection methods rely on single-feature extraction, including optic disc segmentation, cup-to-disc ratio analysis, or standalone texture analysis. While these perform well in some respects, they do not capture the full range of retinal texture and intensity present in fundus images. Also, many studies use limited validation methods and complex DL models, which, in turn, reduce interpretability and robustness in real-world clinical settings. Thus, we see a need for a compute-friendly, highly reliable glaucoma detection system that integrates texture- and intensity-based retinal information and supports robust performance evaluation. This prompted the development of the hybrid LBP-PDC framework we proposed, which we tuned with boosting classifiers and cross-validation to improve glaucoma identification.

### Objectives

The objective of this study is to compare the performance of various models in detecting glaucoma from fundus images and to propose a model with improved performance. This study also aims to analyze the performance of the boosting algorithms on the fundus images dataset and to tune the hyperparameters using various techniques. The methods used for feature extractions are LBP, PDC, and the proposed method (LBP* +*PDC). After feature extraction, the features are used to train several classification models, and their performance is compared using different metrics. Hence, the retrieved features are utilized to categorize fundus images as glaucomatous or non-glaucomatous.

Glaucoma is quite difficult to detect based on a single feature^3^, such as the cup-to-disc ratio or the optic disc radius. In this study, we have extracted features from the entire image using the LBP method. This method considers the complete texture of the image and returns a feature vector. LBP aims to provide an accurate description of the texture of the entire eye and thus will prevent errors that may arise from misinterpreting a few features. The PDC method takes the intensity of white pixels into consideration^4^. Due to the accumulation of intraocular fluid, the optic disc region clouds, and in Grayscale images, this accumulation can be visualized as a white mass. Thus, taking white pixel intensities into account and then calculating the fractal dimension can provide accurate results for glaucoma detection. The proposed model is a hybrid of LBP and PDC and aims to provide improved performance over the other two methods.

Hyperparameter tuning helps us to optimize our models. In this study, the performance of the boosting algorithms is compared by tuning their parameters. GridSearchCV, RandomisedSearchCV, GA, and Bayesian Optimization techniques have been used to tune the hyperparameters. Hyperparameter tuning for CatBoost and Light Gradient Boosting has been performed using the above techniques to obtain different sets of optimized parameters. Thus, this study aims to provide a model for glaucoma detection with improved accuracy and to analyze the performance of boosting algorithms in detecting glaucoma from fundus images.

This research work adds greatly to the field of glaucoma classification and detection. The major contributions made by this research are summarized as below:

- This research gives an important review of glaucoma classification, which can help in gaining insight about how this classification can be carried out and what the drawbacks of the present techniques are.
- This research utilizes the LBP and PDC technique for extraction of the features from the fundus image. With the help of this technique, a feature vector is created for each fundus image.
- The research incorporates a hybrid scheme where features are extracted using both LBP and PDC approaches. The advantage of this scheme is that it incorporates the strengths of the two schemes hence increasing feature representation.
- Hyperparameter tuning for the LightGBM and CatBoost algorithms will be done by the use of several optimization schemes such as GridSearchCV, RandomizedSearchCV, GA, and Bayesian optimization, which will help to optimize the performance of the algorithms by selecting the best hyperparameters.
- Various state-of-the-art classification algorithms such as SVM, DT, RF, KNN, AdaBoost, Gradient Boost, Light gradient boosting, and CatBoost will be evaluated and compared. Such an analysis provides information about the strengths and weaknesses of each algorithm, thus highlighting the importance of the proposed algorithms in glaucoma classification.
- The study gives the results obtained through the 5-fold cross-validation scheme, where apart from the results, many other performance metrics such as accuracy, precision, recall, specificity, f1 score, Matthew’s correlation coefficient, and AUC are given.
- The study results indicate that the proposed framework has been applied to perform comparisons between its performance and those of existing ML and DL based glaucoma detection solutions.

In doing so, the study contributes to glaucoma detection through innovative approaches in features extraction, fine-tuning algorithms for better performance through hyperparameter tuning, and comparison of different classification algorithms. The outcomes of this study could be useful in increasing the efficiency of glaucoma detection systems that could benefit patients as well as physicians in their efforts to diagnose the disease.

### Organization of the paper

The organization of this paper is as follows: In section “Literature survey”, we have provided a comprehensive survey of existing glaucoma detection approaches and relevant literature. In section “Proposed work”, we provide an explanation of our proposed hybrid glaucoma detection approach, which consists of several elements such as preprocessing, feature extraction based on LBP and PDC, feature fusion, various classifiers, and hyperparameter tuning approaches. In section “Results and discussions”, we introduce the experiment setup, including 5-fold cross-validation approach, performance metrics, and comparison of many machine learning algorithms. In section, we provide the results we achieved along with performance comparison and in section “Conclusion and future scope”, we conclude the paper and outline future research directions.

## Literature survey

Various recent literature on artificial intelligence-assisted glaucoma detection demonstrates a clear transition from conventional image-classification pipelines toward clinically interpretable, multi-modal, and deployment-oriented diagnostic systems. Jalili et al. (2025)^18^ evaluated GPT-4 V for fundus-image-based glaucoma assessment and showed that a general multimodal large language model could identify glaucoma-related features such as cup-to-disc ratio, rim thinning, peripapillary atrophy, and image gradability. However, GPT-4 V’s diagnostic accuracy was lower than expert graders across ACRIMA, ORIGA, and RIM-ONE, and approximately 35% of cases required repeated prompt submissions, indicating that current general-purpose vision-language models are not yet reliable substitutes for expert clinical assessment. In contrast, Akter et al. (2025)^2^ focused on functional testing by using Humphrey visual-field pattern deviation plots with ResNet18 and VGG16 to classify glaucoma severity. Their ResNet18 model trained on balanced augmented data achieved a high F1-score, suggesting that visual-field images can support automated staging, although dataset size, imbalance, and limited external validation remain concerns.

Chavan and Choubey (2025)^5^ followed the most common research direction for Fundus-image-based DL and introduced GlaucoAttent Net, a cascaded U-Net with attention mechanisms for optic disc and optic cup segmentation followed by severity classification. The study reported very high diagnostic performance and emphasized the value of combining segmentation-derived anatomical features with classification for clinically meaningful glaucoma grading. Similarly, Gandhi et al. (2025)^6^ proposed a hybrid VGG16 framework in which VGG16 extracted deep features and classifiers such as SVM, RF, and gradient boosting performed the final classification. The VGG16–SVM model achieved strong accuracy, sensitivity, specificity, and interpretability, but the authors noted limitations related to dataset size, demographic imbalance, and computational overhead. Mouhafid et al. (2025)^9^ advanced the lightweight-model direction through SqueezeNet-TR Lite, combining precise optic disc localization, grouped convolutions, transformer encoders, class-balancing strategies, and saliency maps to improve both efficiency and interpretability for large-scale screening.

Sivakumar and Penkova (2025)^11^ proposed multi-modal glaucoma detection by combining retinal images with clinical biomarkers using transformer–CNN hybrid models, with the Swin Transformer–ResNet model achieving the best performance. Gandhi et al. (2025)^7^ further extended this direction by integrating fundus imaging, OCT scans, clinical biomarkers, CNNs, vision transformers, and quantum-enhanced layers. Their framework achieved high accuracy while reducing computational complexity, but they emphasized that performance depends on dataset quality, population diversity, multi-center validation, and explainability. These studies indicate that single-modality fundus analysis may be insufficient for robust clinical diagnosis, especially because glaucoma diagnosis often requires structural, functional, and clinical evidence.

Sharma et al. (2025)^10^ developed AI-GS, a hybrid multi-model fundus-image screening system composed of six lightweight DL models capable of identifying early structural glaucoma signs such as disc cupping, haemorrhage, and retinal nerve fibre layer defects. Their findings are important because the standalone binary classifier showed reduced sensitivity in real-world testing, whereas the full AI-GS network retained better clinical performance. Zhou et al. (2025)^16^ proposed Multi-Glau, a three-tier AI solution aligned with China’s hierarchical healthcare system. It supports primary-care screening without imaging, secondary-care pre-diagnosis with incomplete data, and tertiary-care severity diagnosis using richer clinical inputs. This work is significant because it shifts glaucoma AI from a single diagnostic model to a scalable healthcare-delivery framework, although validation beyond East Asian populations and other healthcare systems remains necessary. Tabei and Askarian (2025)^12^ presented a smartphone-based mHealth approach using CIE LAB enhancement, 3D stereo depth mapping, cup-to-disc ratio extraction, and SVM classification, demonstrating the feasibility of mobile glaucoma screening while noting the need for larger datasets and more robust annotation strategies.

Yadav et al. (2025)^15^ reviewed ML, DL, and hybrid image-analysis techniques for glaucoma detection, highlighting commonly used anatomical features such as cup-to-disc ratio, optic disc, optic cup, retinal nerve fibre layer, peripapillary atrophy, vasculature shift, and neuroretinal rim notching. Yadahalli et al. (2025)^14^ compared multiple DL algorithms and reported that Bilateral U-Net performed strongly across DRISHTI-GS, ORIGA, and ACRIMA, while recommending dataset expansion and architectural improvement for practical clinical use. Gupta and Dada (2025)^8^ emphasized that AI-enabled glaucoma care must address not only model accuracy but also primary-care deployment, LLM hallucination, image quality, real-world validation, cost-effectiveness, and risk of overdiagnosis.

Nam et al. (2024)^19^ demonstrated that optic-disc tilt significantly affects DL-based optic disc classification. Their study showed that VGG16, VGG19, and DenseNet121 models performed better on non-tilted discs than tilted discs, indicating that anatomical variation can reduce diagnostic reliability and must be explicitly considered during model development. Similarly, Chang et al. (2023)^22^ proposed a GAN-based glaucoma detection system that generated OCT-like images from fundus photographs and used incremental learning to adapt the model to different clinical settings. This approach is important because it attempts to overcome the high cost and limited availability of OCT equipment, especially in smaller clinics.

Raza et al. (2023)^25^ created FEDS-Net, a dense segmentation network for optic cup and optic disc segmentation that is based on feature excitation. With just 2.73 million trainable parameters, the model achieved good segmentation performance using feature excitation, information aggregation, fast feature down sampling, and effective convolutional depth, making it highly efficient for screening assistance. For automated detection of glaucoma using optic disc and optic cup localization, Nawaz et al. (2022)^30^ suggested an EfficientDet-D0 framework with an EfficientNet-B0 backbone. Their model outperformed various comparative methods while cutting inference time, achieving an AUC of 0.979 as well as recall of 0.970. Additionally, Singh et al. (2023)^32^ concentrated on optic-cup segmentation utilizing an optimized CNN with the Self-Adaptive Butterfly Optimization Algorithm, morphological and non-morphological extraction of features, modified level set segmentation, and Gaussian filtering.

Several papers extend glaucoma detection beyond simple binary classification. Akter et al. (2022)^28^ performed multi-feature glaucoma diagnosis by combining structural, functional, demographic, and risk-factor data with DL and logistic regression. Their DL model achieved AUC 0.98 and 96% test accuracy, while a pilot OCT-based optic nerve head cup area model achieved AUC 0.99 and 98.6% accuracy, showing that multi-feature and OCT-derived features can improve diagnostic decision support. Thanki (2023)^26^ proposed a deep neural network and ML approach for retinal fundus image classification, where deep features were extracted from fundus images and classified using ML classifiers; the DNN plus logistic regression model showed superior accuracy, sensitivity, and specificity for glaucomatous image classification. These studies suggest that glaucoma AI systems are moving from end-to-end black-box classification toward clinically meaningful feature-based and multi-feature diagnostic models.

The broader medical-image AI papers add useful methodological lessons. Agarwal et al. (2024)^20^ proposed MultiFusionNet for chest X-ray classification by fusing multilayer features from ResNet50V2 and InceptionV3, achieving high accuracy for two-class and three-class disease classification and showing the value of layer-wise feature fusion in limited medical datasets. Ying et al. (2023)^27^ addressed noisy labels in COVID-19 chest X-ray classification using SLIPR, a subset label iterative propagation and replacement algorithm that extracts global and local features using PCA, low-rank representation, neighborhood graph regularization, and KNN before relabelling noisy samples; the method improved classification accuracy across three public datasets but still requires further study under real-world, unpredictable label noise. Malik et al. (2023)^24^ proposed DMFL_Net, a federated learning framework for COVID-19 and multi-chest-disease classification from X-rays, showing that collaborative training can preserve privacy while achieving 98.45% accuracy, though the authors noted that temporal demographic or radiographic shifts were not evaluated.

In cancer-related imaging, Avcı and Karakaya (2023)^21^ developed a mammography enhancement and classification pipeline using median filtering, CLAHE, unsharp masking, k-means clustering, texture feature extraction, and classical ML classifiers. Their results showed that preprocessing combinations strongly influence classification performance, particularly for benign/malignant discrimination. Ayana et al. (2022)^29^ proposed multistage transfer learning for ultrasound breast cancer classification by transferring knowledge from ImageNet to cancer cell-line microscopy images and then to ultrasound breast images. This strategy achieved 99% accuracy on the Mendeley dataset and 98.7% on the MT-Small-Dataset, suggesting that intermediate medical-domain transfer can reduce the need for large ultrasound datasets. Kurdi et al. (2023)^23^ applied Harris Hawks optimized CNN for brain tumour classification from MRI, combining preprocessing, fuzzy/region-growing segmentation, feature extraction, and CNN classification optimized by a metaheuristic algorithm. Finally, Nayak et al. (2022)^31^ proposed a CNN-based brain tumour classification framework with parametric optimization using metaheuristic algorithms. The study used brain MRI images and applied a supervised ResNetV2-based DL model for classifying tumour and healthy MRI images. To reduce training loss and improve model optimization, the authors compared three metaheuristic techniques: Sunflower Optimization Algorithm (SFOA), Forensic-Based Investigation Algorithm (FBIA), and Material Generation Algorithm (MGA). Their experiments were conducted using Taguchi’s L9 design, varying training size, learning rate, and number of epochs. The model reportedly achieved 100% accuracy across nine simulation runs, while SFOA and MGA produced better optimization results than FBIA, whose negative response value made it unsuitable for the considered dataset (Table 1).

**Table 1:** Literature survey of previous work done on biomedical image classifications.

Year & Reference	Title of the paper	Work done	Limitations of the work
2025^2^	Glaucoma Detection and Staging from Visual Field Images Using Machine Learning Techniques	Akter et al. (2025) employed numerical visual-field data and Humphrey 24–2 visual-field pattern deviation graphs	Small and imbalanced dataset; limited external validation; image data were not publicly shareable
2025^5^	Glaucoma Detection and Severity Classification Based on GlaucoAttent Net Framework	GlaucoAttent Net, a cascaded U-Net with focus on optic disc and optic cup segmentation as well as severity classification using CDR and associated anatomical features spanning ORIGA, REFUGE, and G1020, was proposed by Chavan and Choubey (2025)	Requires further validation for clinical generalizability; performance may depend on dataset quality, segmentation accuracy, and computational complexity
2025^6^	Identifying Glaucoma with Deep Learning by Utilizing the VGG16 Model for Retinal Image Analysis	Gandhi et al. (2025) created a hybrid VGG16–ML model that uses SVM/RF/GB classifiers for glaucoma categorization and VGG16 for feature extraction	Computational overhead, demographic imbalance, and limited dataset size
2025^7^	Computational Modeling and Optimization of Deep Learning for Multi-Modal Glaucoma Diagnosis	Gandhi et al. (2025) proposed a multi-modal CNN–ViT– quantum-enhanced framework integrating fundus images, OCT scans, and clinical biomarkers	Model performance depends on dataset diversity and quality. Multi-center validation, lightweight deployment, demographic fairness, and explainability using SHAP, Grad-CAM, or LIME remain necessary
2025^9^	Boosted Diagnostic Accuracy in Glaucoma Detection with SqueezeNet-TR Lite Architecture and Precise Optic Disc Localization	Mouhafid et al. (2025) proposed SqueezeNet-TR Lite with grouped convolutions, simplified fire modules, transformer encoders, optic disc localization, SMOTE-TOMEK balancing, and saliency maps for efficient glaucoma detection	Performance may be affected by imaging variability, fundus quality, class imbalance, and dataset-specific characteristics
2025^10^	A Hybrid Multi Model Artificial Intelligence Approach for Glaucoma Screening Using Fundus Images	Sharma et al. (2025) developed AI-GS, a six-model lightweight screening network for detecting early glaucoma-related signs	Real-world sensitivity dropped for the standalone classifier
2025^11^	Enhancing Glaucoma Detection Through Multi-Modal Integration of Retinal Images and Clinical Biomarkers	Sivakumar and Penkova (2025) integrated retinal images and clinical biomarkers using ViT– ResNet, OWL-ViT–ResNet, and Swin Transformer–ResNet hybrid models	Requires prospective clinical validation, broader external testing, and assessment of cost-effectiveness and accessibility in routine care
2025^12^	A Mobile Health Approach for Glaucoma Detection	Tabei and Askarian (2025) proposed smartphone-based glaucoma detection using CIE LAB enhancement, 3D stereo depth mapping, optic disc/cup extraction, CDR calculation, and SVM classification	Dataset size was limited, and manual annotations may introduce variability. The system needs larger, diverse clinical datasets and stronger validation in real mobile-health settings
2025^13^	A Deep Semi-Supervised Learning Approach to the Detection of Glaucoma on Out- of-Distribution Retinal Fundus Image Datasets	Wang et al. (2025) used semi- supervised learning to grade glaucoma from fundus images with limited labelled data and large-scale unlabelled data	Performance improved with more labelled data but degraded when poor-quality or out-of- distribution unlabelled images were added. Generalization across public datasets remains challenging
2025^14^	Detection Glaucoma Using Deep Learning Algorithms	Yadahalli et al. (2025) compared DL models for glaucoma detection using fundus images	Future work requires dataset expansion, architectural refinement, and practical clinical validation
2025^16^	A Three-Tier AI Solution for Equitable Glaucoma Diagnosis Across China’s Hierarchical Healthcare System	Zhou et al. (2025) proposed Multi-Glau, a three-tier AI system for primary screening, secondary pre-diagnosis with incomplete data, and tertiary severity diagnosis. The system supports resource-limited settings and offline CPU deployment	Focused mainly on glaucoma and East Asian PACG populations; needs validation in diverse global populations, additional chronic eye diseases, and broader public-health equity frameworks
2025^17^	A Generalised Computer Vision Model for Improved Glaucoma Screening Using Fundus Images	Chaurasia et al. (2025) developed a generalized fundus- image screening model and evaluated external performance on Drishti-GS	External validation performance declined compared with internal testing, suggesting dataset inconsistency and the need for larger, more diverse, real-world validation
2025^18^	Glaucoma Detection and Feature Identification via GPT-4 V Fundus Image Analysis	Jalili et al. (2025) evaluated GPT-4 V on 300 fundus images from ACRIMA, ORIGA, and RIM-ONE	The study used only fundus images and lacked OCT, visual- field, and demographic diversity; model behavior may also change with future software updates
2024^19^	Deep learning-based optic disc classification is affected by optic-disc tilt	Nam et al. (2024) studied the effect of optic disc tilt on DL- based optic disc appearance classification using fundus photographs	Classification performance was lower in tilted discs than non- tilted discs; dataset was limited; fundus image heterogeneity may affect performance
2024^20^	MultiFusionNet: multilayer multimodal fusion of deep neural networks for chest X-ray image classification	Agarwal et al. (2024) proposed a multilayer multimodal fusion model using ResNet50V2 and InceptionV3 for chest X-ray classification	Performance depends on dataset quality and transfer learning; broader external validation across institutions is still required
2023^21^	A Novel Medical Image Enhancement Algorithm for Breast Cancer Detection on Mammography Images Using Machine Learning	Avcı and Karakaya (2023) evaluated preprocessing combinations such as median filtering, CLAHE, and unsharp masking for mammography enhancement	Used only 322 mammography images from mini-MIAS; breast cancer type/phenotype information was unavailable
2023^22^	A Glaucoma Detection System Based on Generative Adversarial Network and Incremental Learning	Chang et al. (2023) used GANs to generate OCT-like images from fundus photographs and applied incremental learning so that the model could be fine- tuned with a small number of local clinic or hospital images	Classification accuracy remained moderate.
2023^23^	Brain Tumor Classification Using Meta-Heuristic Optimized Convolutional Neural Networks	Kurdi et al. (2023) proposed a Harris Hawks optimized CNN framework for brain tumor classification from MRI	Evaluated mainly on Kaggle MRI data.
2023^24^	DMFL_Net: A Federated Learning-Based Framework for the Classification of COVID-19 from Multiple Chest Diseases Using X-rays	Malik et al. (2023) developed a federated learning framework using DenseNet-169 to classify COVID-19 while preserving data privacy across clients	Did not investigate temporal changes, demographic shifts, or radiographic drift that may bias diagnostic models
2023^25^	Assisting Glaucoma Screening Process Using Feature Excitation and Information Aggregation Techniques in Retinal Fundus Images	Raza et al. (2023) proposed FEDS-Net for optic cup and optic disc segmentation	Performance depends on fundus image quality, OC/OD boundary clarity, and dataset diversity
2023^26^	A deep neural network and machine learning approach for retinal fundus image classification	Thanki (2023) proposed a computer-aided triage system for glaucoma classification from retinal fundus images	May be sensitive to dataset characteristics and model complexity
2023^27^	COVID-19 chest X-ray image classification in the presence of noisy labels	Ying et al. (2023) proposed SLIPR, a noisy-label recovery algorithm using subset label iterative propagation and replacement	Used random noise simulation; real-world noisy labels may be unpredictable
2022^28^	Glaucoma diagnosis using multi- feature analysis and a deep learning technique	Akter et al. (2022) combined structural, functional, demographic, and risk-factor feature for glaucoma diagnosis	Dataset size was limited
2022^29^	A Novel Multistage Transfer Learning for Ultrasound Breast Cancer Image Classification	Ayana et al. (2022) introduced multistage transfer learning by first transferring ImageNet knowledge to cancer cell-line microscopy images and then to ultrasound breast cancer images	Only three models and three optimizers were tested; hyperparameters were fixed; only two public ultrasound datasets were used
2022^30^	An Efficient Deep Learning Approach to Automatic Glaucoma Detection Using Optic Disc and Optic Cup Localization	Nawaz et al. (2022) proposed EfficientDet-D0 with EfficientNet- B0 backbone for glaucoma localization and classification from fundus images	Performance depends on fundus image quality and dataset variation
2024^31^	Brain Tumour Classification Using Noble Deep Learning Approach with Parametric Optimization through Metaheuristics Approaches	Nayak et al. (2022) proposed a CNN/ResNetV2-based brain tumour classification model using MRI images	The dataset was small, with only 253 MRI images. FBIA produced an infeasible negative response value, making it unsuitable for the considered dataset

A study of works on biomedical image classification using ML and DL techniques was conducted, as shown above. Various feature extraction methods, such as PCA, GLCM, GLRLM, LBP, Pixel Range Calculation, and others like the PDC method, have been used to classify biomedical images, including chest X-ray, fundus, mammogram, and brain MRI images. Various modifications and optimizations have been applied to CNNs for breast cancer, brain tumour, and glaucoma detection. The PDC is a recently developed method for detecting COVID-19 in chest X-ray images, achieving 96% accuracy. In this study, the PDC method is used in combination with the LBP for detecting glaucoma in fundus images.

## Proposed work

### Proposed framework overview

Proposed is a glaucoma detection framework that includes LBP and PDC feature extraction methods for better classification of fundus images. We combine retinal texture information obtained via LBP with white-pixel intensity information from PDC to form a hybrid feature set. We then use this hybrid feature set with multiple ML and boosting algorithms, which we fine-tune via hyperparameter optimization to increase accuracy, robustness, and reliability.

### Dataset description

The ORIGA dataset^33^, a collection of retinal (fundus) images, will be used in this research; these images also include information about the optic nerve head. Fundus images are useful for diagnosing diseases that affect the optic nerve because they allow doctors to observe many important aspects of the retina by using various aspects of these images. Because of its imbalanced nature, the dataset requires robust evaluation of glaucoma classification models. ORIGA was selected as the primary dataset because it is a widely used public benchmark for glaucoma classification and contains retinal fundus images with optic nerve head information relevant to glaucoma assessment. The dataset provides 650 labelled fundus images, including 517 healthy and 133 glaucomatous images, making it suitable for evaluating binary glaucoma classification models under class imbalance. Using a single benchmark dataset also allowed a controlled evaluation of the proposed LBP-PDC feature extraction framework without introducing dataset-level variations caused by different acquisition devices, image resolutions, annotation protocols, and population characteristics.

Preprocessing consists of resizing fundus images to the same dimension and converting them to grayscale before extracting features. All images are processed using the proposed feature extraction methods to generate feature vectors for training the classification models. Using a 5-fold cross-validation process improves robustness and reduces bias during model evaluation for the proposed framework.

### Feature extraction

The process of extracting features from images involves considering certain characteristics of the images. A comparison of the features extracted from various images can help us to identify glaucomatous eyes. Various features that can be considered for detecting glaucoma include optic cup and optic disc dimensions, the cup-to-disc ratio, retinal thickness, and more. The images of eyes not affected by glaucoma show features similar to those of healthy eye images, whereas glaucoma-positive images differ from those of normal eye images. Here, we have used the LBP and PDC methods for feature extraction.

The LBP technique is used to identify retinal textures, and the PDC is used for structure-based analysis of image intensity. When we combine both, we obtain a hybrid feature that, in turn, improves glaucoma classification performance compared with using either technique alone. feature extraction techniques.

#### Local Binary Pattern (LBP)

LBP is a feature extraction method used to obtain a texture description of the entire image^15^. To apply the LBP method, we first need to convert the image to grayscale. There are mainly two parameters which are used in the LBP method: (i) the number of neighbours $\:\left(n\right)$ to be considered, and (ii) the radius of the neighbourhood $\:\left(r\right)$. Here, we have taken * n=8* and * r=1*. Thus, a $\:3\times\:3$ neighbourhood window is considered, and pixel intensities are calculated for all 9 blocks in the grid.

The threshold is the pixel intensity of the grid’s center pixel. Every adjacent pixel’s intensity is contrasted with the central pixels. We assign 1 if the neighboring pixel value is higher than the center value, and 0 if it is lower than or equal to the center value. A binary number is obtained by comparing the nearby pixels in a clockwise direction and storing their results in an array. An LBP code is created by converting the binary code to decimal form. The computed LBP value is substituted for the central pixel.

1 $$LBP_{{n,r}} = \mathop \sum \limits_{{i = 0}}^{{n - 1}} f\left( x \right){ { \times}}2^{i}$$

2 $$\:f\left(x\right)=\left\{\begin{array}{c}1\:\:if\:x\ge\:0\\\:0\:if\:x<0\end{array}\right.\:\:\:\:\:\:\:\:where\:x=\:{pi}_{n}-\:{pi}_{c}$$

Here, $\:{LBP}_{n,r}$ is the LBP code calculated for the $\:{i}{th}$ neighboring pixel, and * n* is the number of neighbors, and * r* is the radius of neighborhood, and $\:{pi}_{n}$ is the pixel intensity of the corresponding neighboring pixel and $\:{pi}_{c}$ is the pixel intensity of the central pixel.

Similarly, this process is repeated across the entire image, and an LBP code is calculated for each pixel. Thus, the LBP code is obtained for the entire image. Various texture patterns are obtained from the entire image. Based on the different texture patterns obtained, histograms are constructed, with each bin representing one texture pattern. The frequency of each texture pattern can be calculated using the histogram. Next, we obtain the feature vector from the histogram. The feature vector is a collection of different features obtained from the image.

**Fig. 1:**
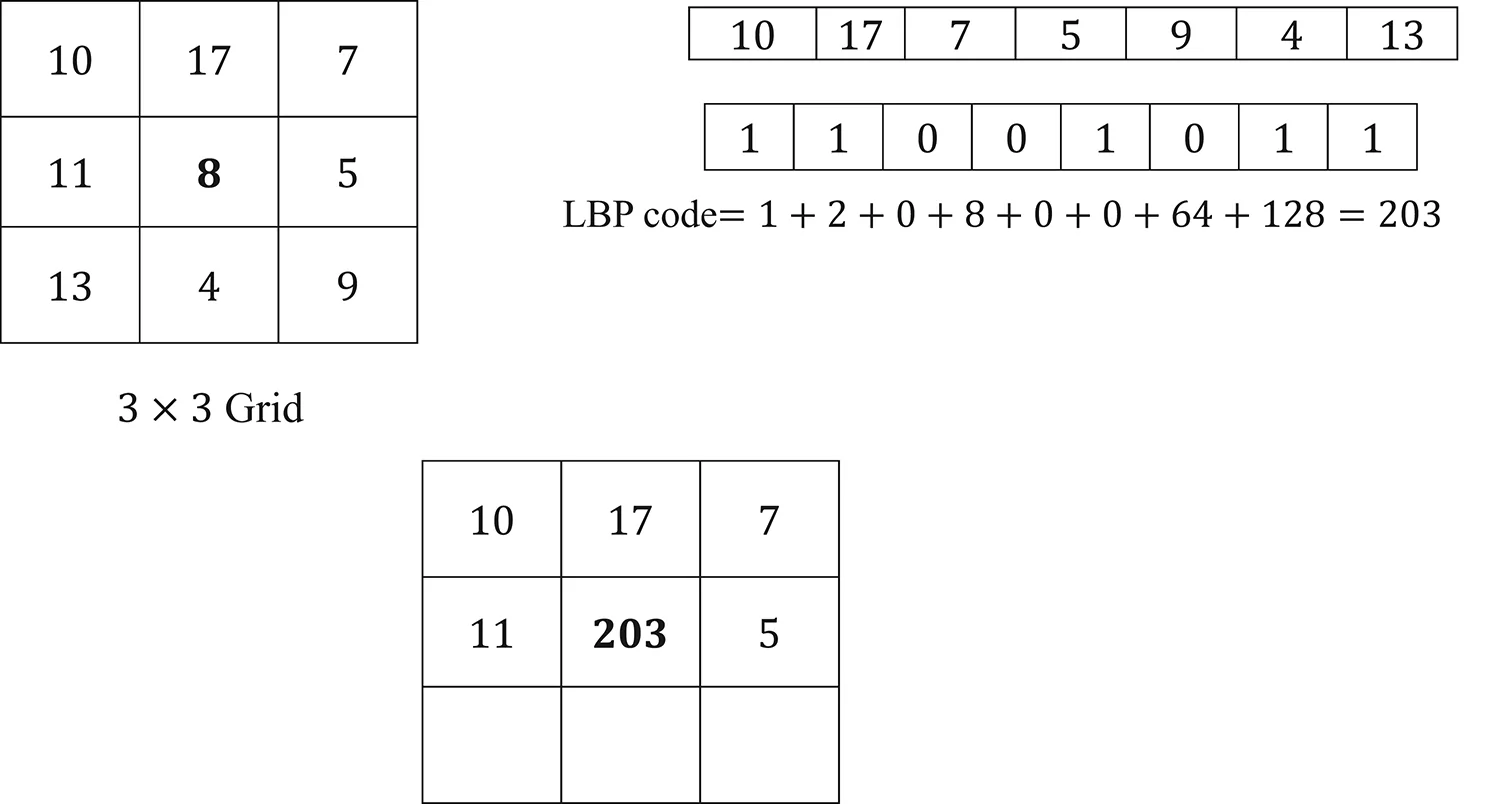
Computation of LBP code for a grid.

The extracted LBP histogram features provide an efficient description of local retinal texture changes, which, in turn, are related to glaucomatous abnormalities. These features are also used for training classification models, which in turn improve glaucoma detection performance (Fig. 1).

In the present implementation, the LBP parameters were fixed at * P=8* neighboring pixels and radius * R=1*, corresponding to a $\:3\times\:3$ local neighborhood. This setting was selected because it captures fine local retinal texture while keeping the feature dimension and computational cost low. However, LBP performance can vary with the radius and number of neighbors; therefore, a sensitivity analysis should be included to evaluate the robustness of the proposed framework to different LBP configurations.

#### Pivot Distribution Count (PDC)

The PDC Method is used to extract white pixel intensity from fundus images^32^. Glaucoma is caused by the accumulation of intraocular fluid in the optic cup region. Thus, this accumulation of fluid can be visualized as clouding of the optic nerve head. This accumulation of fluid can be represented as a white mass in the optic cup region. Thus, counting white pixel intensity in fundus images can be used to detect glaucoma. In the PDC method, the image is first converted to greyscale. The pixel intensities are calculated for all the pixels of the fundus image.

We need to set a threshold for all pixel intensities, and the number of pixels exceeding this threshold will be considered the white pixel count. This threshold value is called as the pivot value. To calculate the pivot value ($\:{P}_{0\:}$) using Eq. (3), we first compute the mean of all pixel intensities in the image ($\:{P}_{mean}$), then the maximum pixel intensity ($\:{P}_{max}$), and finally the mean of $\:{P}_{mean}$ and $\:{P}_{max}$ gives $\:{P}_{0}$.

3 $$\:{P}_{0\:}=\frac{\left({P}_{mean}+\:{P}_{max}\right)}{2}$$

After calculating the pivot value, we compare all pixel intensities to it, and the number of pixel intensities exceeding the pivot value is used as the white pixel count ($\:{{W}_{p}}_{i}$). Thus, the white pixel counts for all the images are calculated. Then, we calculate the fractal dimension of the image by using the white pixel count using the formula below:

4 $$\:{FD}_{i}=\:\frac{\mathrm{log}\left({{W}_{p}}_{i}\right)}{\mathrm{log}\left(\frac{1}{r}\right)}$$

Here, $$\:{FD}_{i}$$ is the fractal dimension for the $$\:{i}{th}$$ image and $$\:{{W}_{p}}_{i}$$ is the white pixel count for the $$\:{i}{th}$$ image, and $$\:\raisebox{1ex}{$1$}\!\left/\:\!\raisebox{-1ex}{$r$}\right.$$ is the factor of reduction calculated using the length of the image and the length of the box. Now, we set a threshold value (* T*) for detecting glaucoma by using the fractal dimension.

5 $$\:T=\frac{\sum\:_{i=0}^{i=n-1}{FD}_{i}}{n}$$

Here, * n* is the total number of images present in the dataset. Now, to detect glaucoma, we compare the image’s fractal dimension against the threshold value. If the fractal dimension of the image exceeds the threshold value, the image is detected as glaucoma-positive; otherwise, it is considered glaucoma-negative.

The PDC approach we see is based on intensity in retinal fundus images. What we get out of it are features that point out white-pixel distribution, which in turn, present glaucomatous abnormalities and which, in turn, improve the feature representation for glaucoma classification.

#### Proposed LBP – PDC method

A hybrid model for image extraction can be formed by combining the LBP and the PDC methods for feature extraction. In the proposed methodology, we first obtain the LBP code for all pixels in the fundus image. At first, the LBP code for the greyscale image is obtained using Eq. (6) below:

6 $$LBP_{{n,r}} = ~\mathop \sum \limits_{{i = 0}}^{{n - 1}} f\left( p \right){{ \times }}2^{i}$$

7 $$\:f\left(p\right)=\left\{\begin{array}{c}1\:\:if\:p\ge\:0\\\:0\:if\:xp<0\end{array}\right.\:\:\:\:\:\:\:\:\:\:\:\:\:\:where\:p={pi}_{n}-\:{pi}_{c}$$

Here, $\:{LBP}_{n,r}$ is the LBP code calculated for the $\:{i}^{th}$ neighboring pixel, * n* depicts the number of neighbors, and * r* is the radius of neighborhood, and $\:{pi}_{n}$ is the pixel intensity of the corresponding neighboring pixel and $\:{pi}_{c}$ is the pixel intensity of the central pixel.

After we obtain the LBP image, we use the PDC method to extract white-pixel-intensity distribution features from it. Thus, for the LBP image, we need to set a threshold value. Pivot value is calculated for each LBP image using the equation below. This threshold value is called the pivot value. In order to calculate the pivot value $\:\left({P}_{0\:\left(lbp\right)}\right)$ using Eq. (3), where $\:{P}_{mean\:\left(lbp\right)}$ is the mean of all pixel intensities of the LBP images and $\:{P}_{max\:\left(lbp\right)}$ is the maximum pixel intensity of the LBP image.

8 $$\:{P}_{0\:\left(lbp\right)}=\frac{\left({P}_{mean\:\left(lbp\right)}+\:{P}_{\mathrm{m}\mathrm{a}\mathrm{x}\left(lbp\right)}\right)}{2}$$

Once the pivot value is calculated, we set it as the threshold, which in turn makes pixels that exceed it appear white in the LBP image. After we have the white-pixel counts for all LBP images, we then calculate the fractal dimension of each LBP image using this Eq. 

9 $$\:{FD}_{{lbp}_{i}}=\:\:\frac{\mathrm{log}\left({{W}_{lbp}}_{i}\right)}{\mathrm{log}\left(\frac{1}{r}\right)}$$

where, $$\:{FD}_{{lbp}_{i}}$$ is the fractal dimension for the $$\:{i}^{th}$$ LBP image and $$\:{{W}_{lbp}}_{i}$$ is the white pixel count for the $$\:{i}^{th}$$ LBP image, and $$\:\raisebox{1ex}{$1$}\!\left/\:\!\raisebox{-1ex}{$r$}\right.$$ is the factor of reduction. After the calculation of the fractal dimension for each LBP image, a threshold value needs to be set using the equation below:

10 $$\:{T}_{lbp}=\frac{\sum\:_{i=0}^{i=n-1}{FD}_{{lbp}_{i}}}{n}$$

where * n* is the total number of images present in the dataset. Finally, we compare the fractal dimension of each LBP image against a threshold. If the fractal dimension exceeds the threshold, the image is classified as glaucoma-positive; if not, it is classified as glaucoma-negative. If the fractal dimension of the LBP image ($\:{FD}_{{lbp}_{i}}$) is greater than the threshold value ($\:{T}_{lbp})$. If calculated, the fundus image is classified as glaucoma-positive; otherwise, it is classified as glaucoma-negative.

**Fig. 2:**
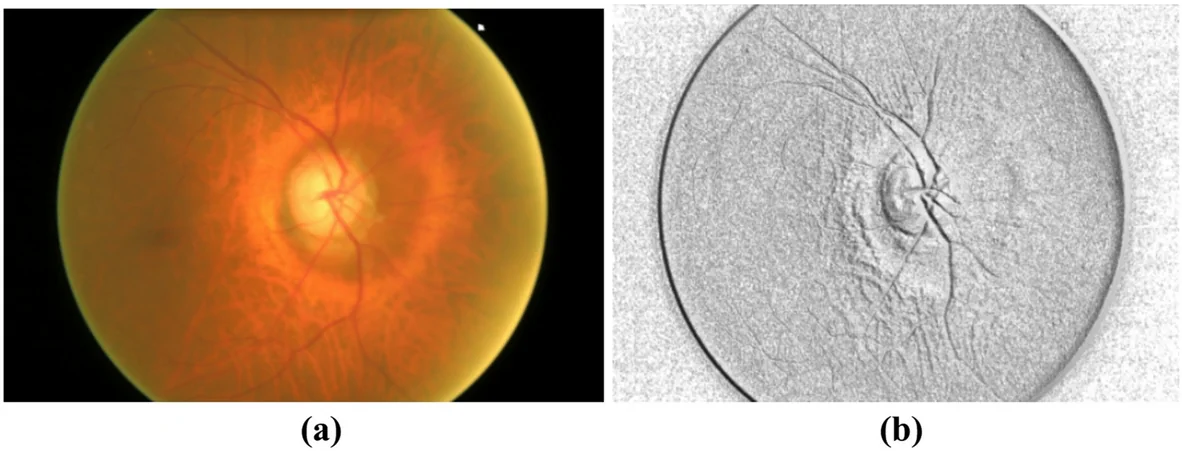
**(a)** Fundus image **(b)** LBP form of the fundus image.

The proposed hybrid LBP-PDC framework performs texture- and intensity-based retinal feature extraction to improve glaucoma detection. While LBP does a good job of capturing local retinal textural variations, PDC collapses this information into a white-pixel-intensity distribution, a characteristic of glaucomatous damage. We see that using both methods yields a much better overall picture and improved classification performance compared to using individual features (Fig. 2).

#### Classification of Fundus Images

The extracted hybrid LBP-PDC feature vectors were used to train various ML and boosting classifiers for glaucoma classification. We evaluated various models to assess the performance and robustness of the proposed feature extraction framework.

After feature extraction, the classification models are trained on these features to perform binary classification of fundus images as glaucomatous or non–glaucomatous. The classification method is a supervised learning technique that uses labelled data to train the model, and the testing data is then used to predict. Thus, the features obtained using LBP, PDC and the proposed method (LBP* +*PDC) are used to train the classification models. The classification methods used here are SVM, Decision Tree, Random Forest, KNN, Adaptive Boosting, Gradient Boosting, XGBoost, Light Gradient Boosting, and CatBoost.

For each classification model, the optimized hyperparameters are obtained using GridSearchCV and RandomizedSearchCV. Table 2 shows the classification methods used and their optimized parameters for classifying features obtained using the LBP method. Table 3 shows the classification methods used and their optimized parameters for classifying features obtained using the PDC method. Table 4 shows the classification methods used and their optimized parameters for classifying features obtained with the proposed method.

To improve the robustness and reduce bias in model evaluation, we used a 5-fold cross-validation strategy. The feature vectors obtained from LBP, PDC, and the proposed hybrid LBP-PDC were used to train and test the classification models.

**Table 2:** Classification methods used and their parameters for LBP.

Methods	Optimized parameters
Decision Tree	criterion=‘gini’, random_state = 0, max_depth = 10, max_features = 1, min_samples_split = 30
Random Forest	criterion=‘gini’, random_state = 0, max_depth = 10, max_features = 1, n_ estimators = 50, min_samples_split = 40
KNN	n_neighbors = 21
SVM	C = 10, kernel=‘poly’, gamma = 0.1
AdaBoost	n_estimators = 100, learning_rate = 0.01, base_estimator = rf, random_state = 10
Gradient Boosting	n_estimators = 100, learning_rate = 0.01, random_state = 10, max_features = 10
XGBoost	learning_rate = 0.001, n_estimators = 100, max_depth = 10, min_child_weight = 2, gamma = 0, subsample = 0.5
LGBM (best)	boosting_type = goss, importance_type = split, learning_rate = 0.559, reg_alpha = 0.894, reg_lambda = 0.135, random_state = 25
CatBoost (best)	learning_rate = 0.05, l2_leaf_reg = 4, iterations = 20, depth = 10, bootstrap_type = ‘Bernoulli’, random_seed = 25

**Table 3:** Classification methods used and their optimized parameters for PDC.

Methods	Optimized parameters
Decision Tree	criterion=‘gini’, random_state = 0, max_depth = 10, max_features = 1, min_samples_split = 20
Random Forest	criterion=‘gini’, random_state = 0, max_depth = 20, max_features = 2, n_estimators = 50
KNN	n_neighbors = 11
SVM	C = 10, kernel=‘poly’, gamma=‘auto’
AdaBoost	n_estimators = 50, learning_rate = 0.001, base_estimator = dt, random_state = 0
Gradient Boosting	n_estimators = 400, learning_rate = 0.1, random_state = 0, max_features = 2
XGBoost	learning_rate = 0.001, n_estimators = 400, max_depth = 20
LGBM (best)	boosting_type = dart, importance_type = gain, learning_rate = 0.086, reg_alpha = 0.718, reg_lambda = 0.449
CatBoost (best)	learning_rate = 0.05, l2_leaf_reg = 4, iterations = 20, depth = 10, bootstrap_type = ‘Bernoulli’ ,random_seed = 30

**Table 4:** Classification methods used and their optimized parameters for proposed work.

Methods	Optimized parameters
Decision Tree	criterion=‘gini’, random_state = 42, max_depth = 10, max_features = 1, min_samples_split = 10
Random Forest	criterion=‘gini’, random_state = 0, max_depth = 20, max_features = 2, n_estimators = 50, min_samples_split = 40
KNN	n_neighbors = 11
SVM	C = 1, kernel=‘rbf’, gamma=‘auto’
AdaBoost	n_estimators = 50, learning_rate = 0.001, base_estimator = dt, random_state = 0
Gradient Boosting	n_estimators = 200, learning_rate = 0.01, random_state = 42, max_features = 1
XGBoost	learning_rate = 0.001, n_estimators = 400, max_depth = 20
LGBM (best)	boosting_type = ‘gbdt’, learning_rate = 0.161, importance_type = ‘split’, reg_alpha = 0.452, reg_lambda = 0.322, random_state = 25
CatBoost (best)	learning_rate = 0.368, iterations = 23, depth = 15, l2_leaf_reg = 3, bootstrap_type= ‘Bayesian’, random_state = 25

### Hyperparameter tuning of LGBM and CatBoost

Hyperparameter tuning is done to improve the performance and robustness of the classification models. We find that selecting optimal hyperparameters improves classification accuracy, reduces overfitting, and enhances the generalization of our proposed glaucoma detection framework.

In this study, we use GridSearchCV and RandomizedSearchCV methods to determine the best hyperparameters for our models. These methods help us find the best parameter set, thereby improving glaucoma classification performance. We present the optimized hyperparameters for each classification model in the respective tables for the LBP, PDC, and hybrid LBP-PDC feature extraction methods.

#### Genetic Algorithm (GA)

In GA, we provide an initial population of information, which is a range of values for the hyperparameters that are to be tuned. This initial information can be considered as an individual of the population. A GA is used to provide accurate solutions to optimization problems. The various operations of the GA are selection, crossover, mutation, and encoding. In the first step of the GA, we create an initial population and then calculate the fitness of the individuals. Then we check whether we have obtained the optimized solution. If the condition is not satisfied, we select some individuals using a tournament, then perform crossover and mutation to find optimized solutions.

#### Bayesian optimization

Bayesian Optimization is a hyperparameter tuning method that learns from the performance of previous hyperparameter combinations. Thus, this gives the optimal set of parameters in less time. In this study, we have used the Hyperopt package available in Python to achieve Bayesian optimization. In this method, there are mainly three operators we need to provide: the parameter space, the objective function, and the selection function. Along with these, the maximum number of evaluations needs to be provided. The parameter space is the set of parameters we need to optimize, along with their ranges. The objective function here is the classification method whose parameters need to be optimized; the purpose is to optimize it, and accuracy is used as the evaluation metric. Algorithm 1 gives the optimized LGBM and CatBoost classification using GA and Bayesian Optimization.

#### Optimized classification workflow

Algorithm1 summarizes the overall experimental pipeline used for optimized glaucoma classification. First, each retinal fundus image is pre-processed by resizing and converting it into grayscale. Then, features are extracted using LBP, PDC, or the proposed hybrid LBP-PDC method. The extracted feature vectors are used as input to the selected classifier.

To obtain robust performance estimates, the dataset is evaluated using 5-fold cross-validation. In each fold, the training data are used to optimize the classifier hyperparameters, while the testing data are used only for performance evaluation. LightGBM and CatBoost are selected for optimization because they showed strong classification performance in the proposed framework. Hyperparameter tuning is performed using GridSearchCV, RandomizedSearchCV, genetic algorithm, or Bayesian optimization.

**Figure:**
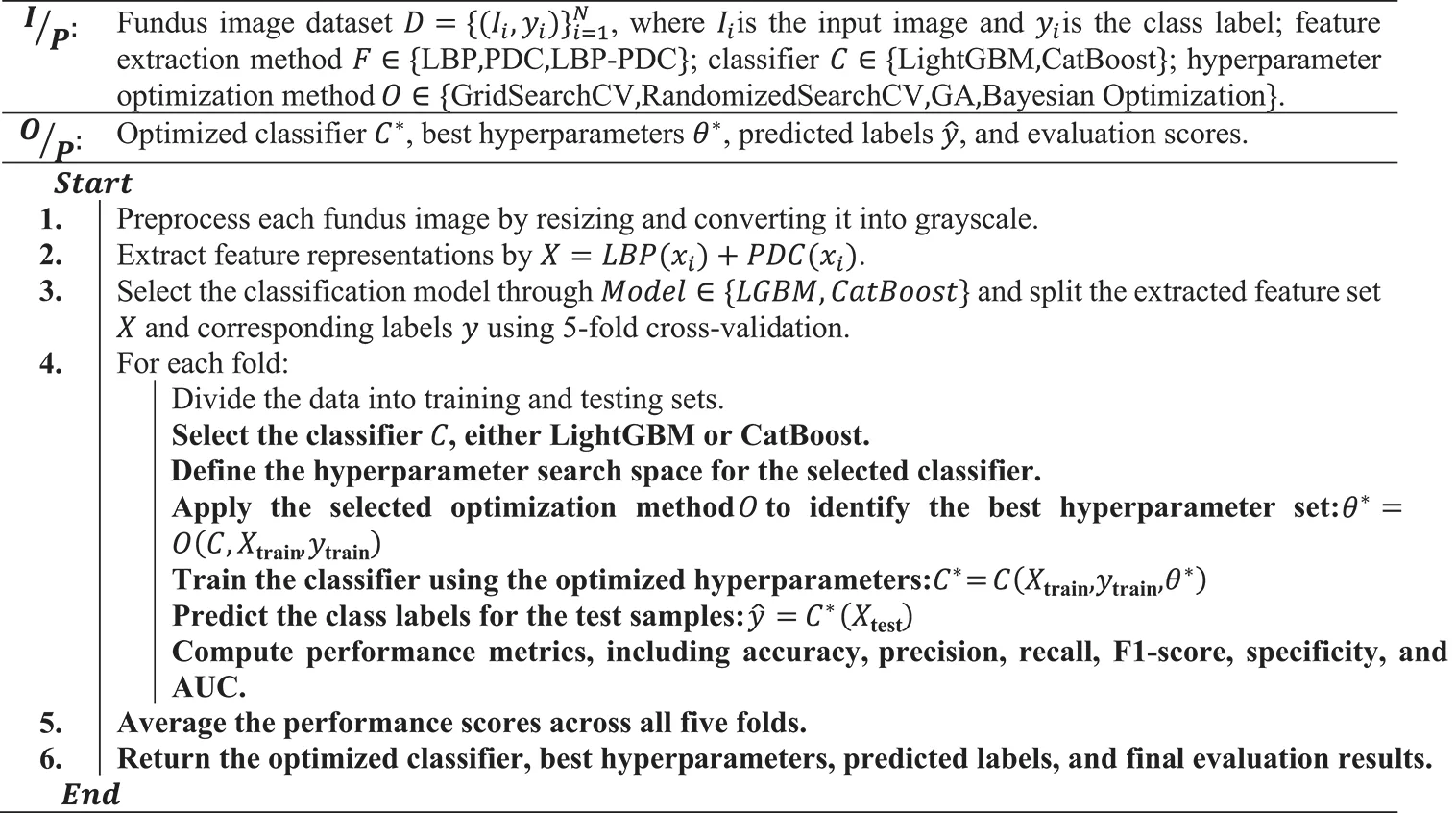
**Algorithm 1** Optimized glaucoma classification using hybrid LBP-PDC features.

This algorithm first extracts LBP, PDC, or LBP* +*PDC features from the input training samples. Then either LGBM or CatBoost are selected as the classifier. The hyperparameters of the selected classifier are optimized using either GA or Bayesian Optimization. After obtaining the optimal hyperparameter set, the final classifier is trained. LGBM uses EFB and GOSS to construct optimized decision trees, while CatBoost uses ordered boosting with permuted training samples and base learners. Finally, the optimized model is used to generate predictions and evaluate classification performance (Tables 5, 6 and 7).

**Table 5:** Optimized parameters after hypertuning of LGBM and CatBoost for LBP.

Methods	Optimized parameters
LGBM* +*GS	boosting_type = gbdt, importance_type = gain, learning_rate = 0.001, reg_alpha = 0, reg_lambda = 0
LGBM* +*RS	boosting_type = dart, importance_type = gain, learning_rate = 0.01, reg_alpha = 1.5, reg_lambda = 2.0
LGBM* +*GA	boosting_type = gbdt, importance_type = gain, learning_rate = 0.598, reg_alpha = 0.753, reg_lambda = 0.430
LGBM* +*Bayesian	boosting_type =darts, importance_type = split, learning_rate = 0.092, reg_alpha = 0.828, reg_lambda = 0.382
CatBoost* +*GS	bootstrap_type = Bernoulli, depth = 10, iterations = 5, l2_leaf_reg = 3, learning_rate = 0.001
CatBoost* +*RS	bootstrap_type = Bernoulli, depth = 10, iterations = 20, l2_leaf_reg = 4, learning_rate = 0.05
CatBoost* +*GA	bootstrap_type = ‘Bernoulli’, depth = 11, iterations = 15, l2_leaf_reg = 2, learning_rate = 0.750
CatBoost* +*Bayesian	bootstrap_type =Bernoulli, depth = 7, iterations = 13, l2_leaf_reg =, learning_rate = 0.219

**Table 6:** Optimized parameters after hypertuning of LGBM and CatBoost for PDC.

Methods	Optimized parameters
LGBM* +*GS	boosting_type = dart, importance_type = gain, learning_rate = 1.0, reg_alpha = 0.01, reg_lambda = 0.1
LGBM* +*RS	boosting_type = dart, importance_type = gain, learning_rate = 0.01, reg_alpha = 0.1, reg_lambda = 1.0
LGBM* +*GA	boosting_type = gbdt, importance_type = split, learning_rate = 0.568, reg_alpha = 0.348, reg_lambda = 0.334
LGBM* +*Bayesian	boosting_type = gbdt, importance_type = gain, learning_rate = 0.547, reg_alpha = 0.610, reg_lambda = 0.333
CatBoost* +*GS	bootstrap_type = Bayesian, depth = 16, iterations = 20, l2_leaf_reg = 3, learning_rate = 0.05
CatBoost* +*RS	bootstrap_type = Bernoulli, depth = 10, iterations = 20, l2_leaf_reg = 4, learning_rate = 0.05
CatBoost* +*GA	bootstrap_type = Bayesian, depth = 10, iterations = 19, l2_leaf_reg = 2, learning_rate = 0.261
CatBoost* +*Bayesian	bootstrap_type = Bernoulli, depth = 7, iterations = 13, l2_leaf_reg = 6, learning_rate = 0.423

**Table 7:** Optimized parameters after hypertuning of LGBM and CatBoost for proposed work.

Methods	Optimized parameters
LGBM* +*GS	boosting_type =‘gbdt’, importance_type = ‘gain’, learning_rate = 0.001, reg_alpha = 0.001, reg_lambda = 0.001
LGBM* +*RS	boosting_type =‘dart’, importance_type = ‘gain’, learning_rate = 0.01, reg_alpha = 0.1, reg_lambda = 1.0
LGBM* +*GA	boosting_type = ‘gbdt’, learning_rate = 0.161, importance_type = ‘split’, reg_alpha = 0.452, reg_lambda = 0.322
LGBM* +*Bayesian	boosting_type = ‘gbdt’, learning_rate = 0.572, importance_type = ‘split’, reg_alpha = 0.272, reg_lambda = 0.787
CatBoost* +*GS	bootstrap_type = ‘Bayesian’, depth = 4, iterations = 10, l2_leaf_reg = 3, learning_rate = 0.001
CatBoost* +*RS	learning_rate = 0.001, l2_leaf_reg = 4, iterations = 10, depth = 16, bootstrap_type =‘Bayesian’
CatBoost* +*GA	learning_rate = 0.368, iterations = 23, depth = 15, l2_leaf_reg = 3, bootstrap_type= ‘Bayesian’
CatBoost* +*Bayesian	bootstrap_type = ‘Bayesian’, depth = 4, iterations = 14, l2_leaf_reg = 2, learning_rate = 0.228

### Performance evaluation

**Fig. 3:**
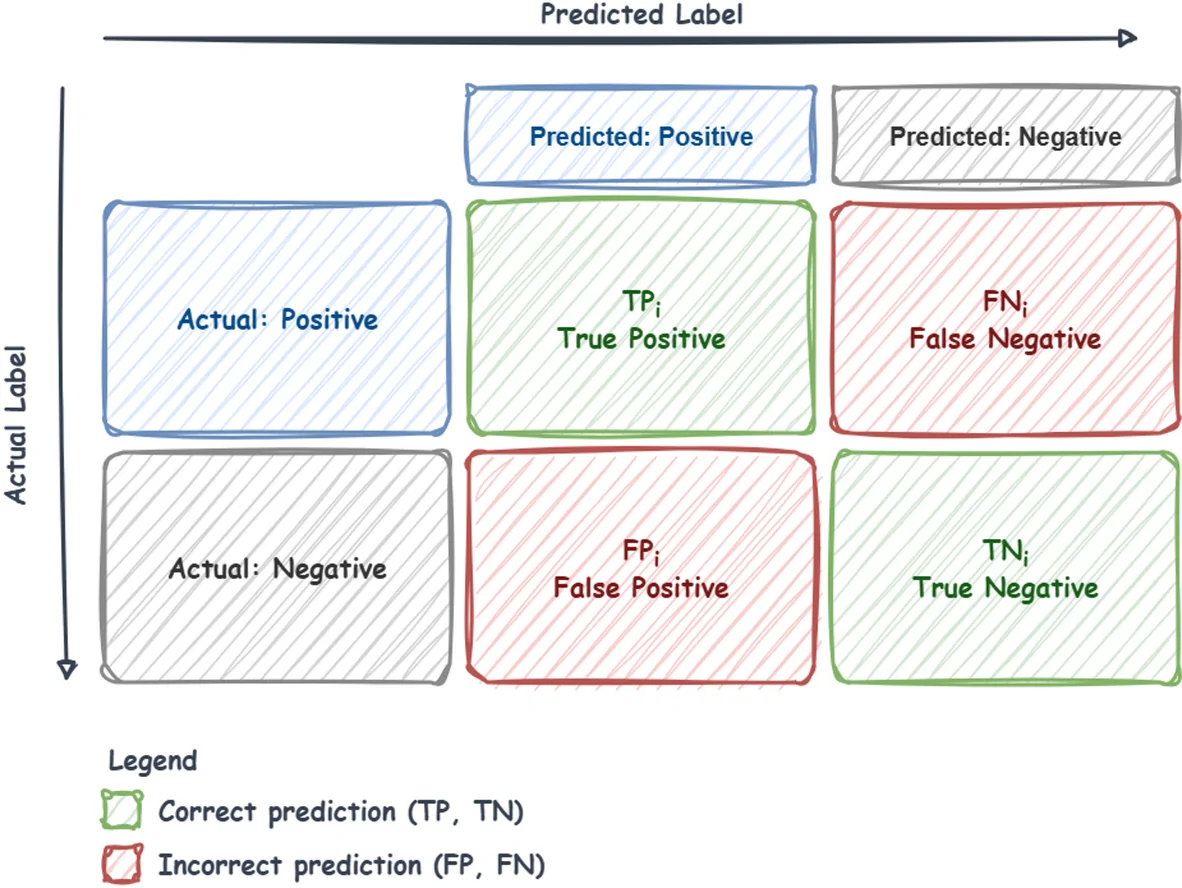
Confusion matrix for binary classification.

**Fig. 4:**
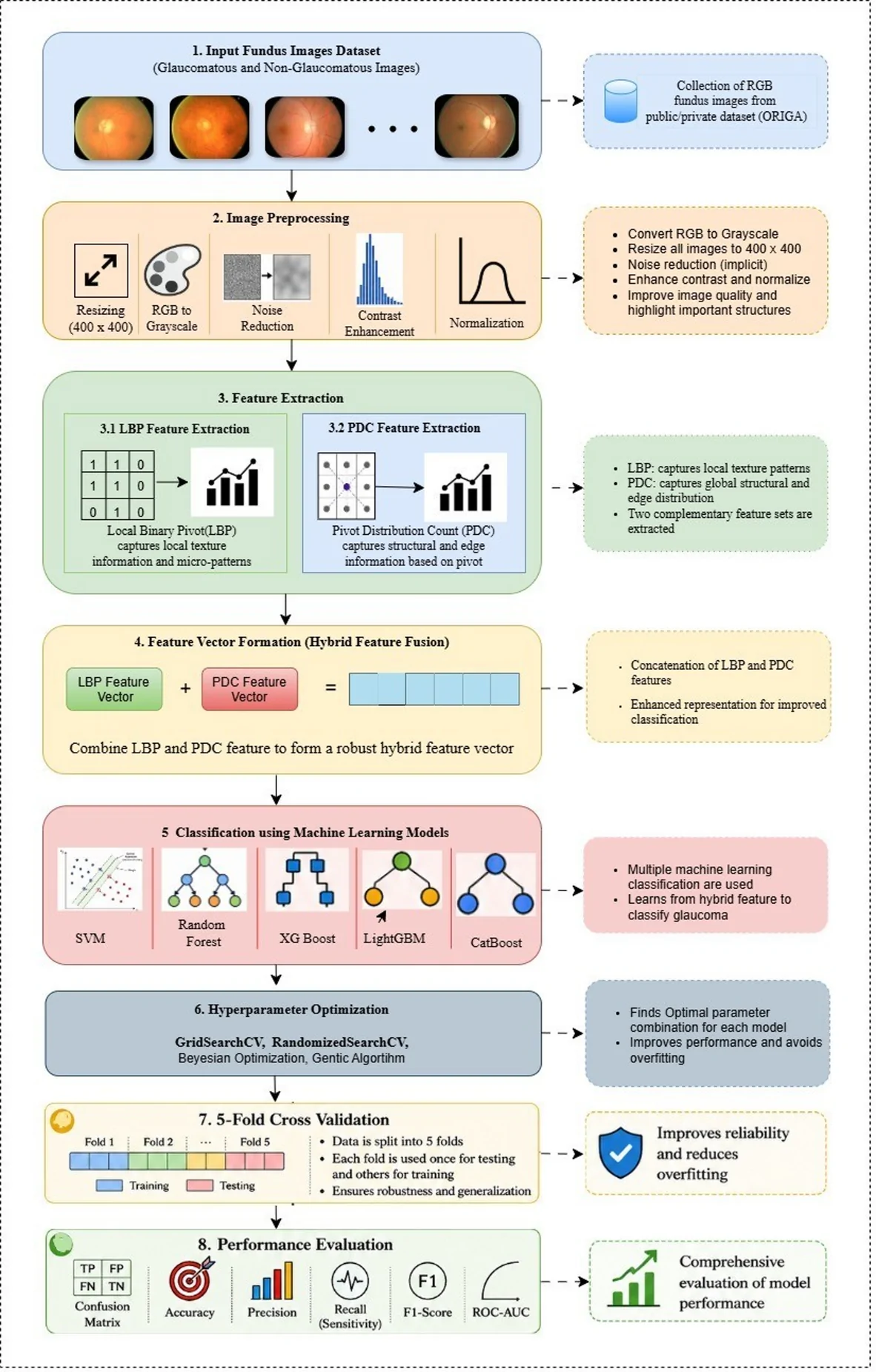
Proposed hybrid LBP-PDC framework for automated glaucoma detection using fundus images.

A confusion matrix is used to evaluate the performance of the model used. For binary classification, a $\:2\times\:2$ confusion matrix is plotted, where the rows represent the actual classes and the columns represent the predicted classes. From the confusion matrix, we can calculate various performance metrics such as positive predictive value, negative predictive value, sensitivity, specificity, accuracy, error rate, recall, precision, and F–measure. Here, we have calculated performance metrics such as accuracy, error rate, precision, recall, and F1-score to evaluate the performance of the models used (Figs. 3, 4).

- True Positive ($\:{TP}_{i}$): It represents the data points that are actually positive and are predicted as positive.
- False Positive ($\:{FP}_{i}$_i_): It represents the data points that are actually negative but are predicted as positive.

- True Negative ($\:{TN}_{i}$): It represents the data points that are actually negative and are predicted as negative.
- False Negative ($\:{FN}_{i}$): It represents the data points that are actually positive and are predicted as negative.

The performance metrics calculated for each model of classification with the help of the confusion matrix and their formulas are mentioned below:

11 $$\:Accuracy=\frac{{TP}_{i}+\:{TN}_{i}}{{TP}_{i}+\:{TN}_{i}+\:{FP}_{i}+\:{FN}_{i}}$$

12 $$\:Error\:rate=\:\frac{{FP}_{i}+\:{FN}_{i}}{{TP}_{i}+\:{TN}_{i}+\:{FP}_{i}+\:{FN}_{i}}$$

13 $$\:Precsion=\:\frac{{TP}_{i}}{{TP}_{i}+\:{FP}_{i}}$$

14 $$\:Recall=\:\frac{{TP}_{i}}{{TP}_{i}+\:{FN}_{i}}$$

15 $$\:F\:score=\:\frac{2\:\times\:Precision\:\times\:Recall}{Precision+\:Recall}$$

## Results and discussions

The confusion matrices for each classification method used have been plotted. For evaluating the models, accuracy, error rate, precision, recall, and f1-score for all the methods are calculated. Our proposed glaucoma detection framework was evaluated using 5-fold cross-validation to ensure reliable and robust model performance. It is found that the method of KNN classification gives the highest accuracy of 79.23% for LBP, and for PDC, the highest accuracy of 95.71% is obtained using SVM, and for the proposed method, Light GBM and CatBoost performed the best with an accuracy of 97.55%. By hypertuning parameters on LGBM and CatBoost, we obtained the highest accuracy of 77.69% with CatBoost using Bayesian optimization and 77.69% with LGBM using a GA. For the PDC method, we have obtained the highest accuracy of 94.48% after hypertuning the parameters of LGBM using all four methods and CatBoost using RandomizedSearchCV. For, the proposed model, accuracy of 97.55% is obtained after hypertuning of LGBM and CatBoost with all four hypertuning methods (GridSearchCV, RandomizedSearchCV, GA and Bayesian Optimization).

Table 8 represents the various performance metrics for all the classification methods used for classifying the fundus images using the features extracted by using the LBP method. Table 9 represents the various performance metrics for all the classification methods used for classifying the fundus images using the features extracted by using the PDC method. Table 10 represents the various performance metrics for all the classification methods used for classifying the fundus images using the features extracted by using the proposed method. Table 11 represents the various performance metrics for the classification methods LGBM and CatBoost using three hypertuning methods GridSearchCV, RandomizedSearchCV, GA and Bayesian Optimization for LBP. Table 12 represents the various performance metrics for the classification methods LGBM and CatBoost using three hypertuning methods GridSearchCV, RandomizedSearchCV, GA and Bayesian Optimization for PDC. Table 13 represents the various performance metrics for the classification methods LGBM and CatBoost using three hypertuning methods GridSearchCV, RandomizedSearchCV, GA and Bayesian Optimization for the Proposed Method.

### Performance metrics tables

**Table 8:** Results for LBP method.

Methods	Accuracy ($\:\%$)	Error rate ($\:\%$)	Precision	Recall	F-score	AUC
Decision Tree	70	30.00	0.27	0.32	0.29	0.548
Random Forest	76.92	23.08	0.27	0.50	0.35	0.523
KNN	**79.23**	**20.77**	**0.13**	**0.80**	**0.23**	**0.562**
SVM	77.69	22.31	0.10	0.60	0.17	0.540
AdaBoost	76.15	23.85	0.07	0.40	0.11	0.518
Gradient Boosting	77.69	23.85	0.03	1.00	0.06	0.517
XGBoost	76.15	22.31	0.07	0.40	0.11	0.530
LGBM (best)	77.69	22.31	0.17	0.56	0.06	0.563
CatBoost (best)	77.69	22.31	0.07	0.67	0.12	0.528

Significant values are in [bold].

**Table 9:** Results for PDC method.

Methods	Accuracy ($\:\%$)	Error rate ($\:\%$)	Precision	Recall	F-score	AUC
Decision Tree	92.64	7.36	0.88	1.00	0.93	0.938
Random Forest	94.48	5.52	0.91	1.00	0.95	0.953
KNN	93.25	6.75	0.89	1.00	0.94	0.943
SVM	**95.71**	**4.29**	**1.00**	**0.93**	**0.96**	**0.948**
AdaBoost	94.48	5.52	0.91	1.00	0.95	0.953
Gradient Boosting	94.48	5.52	0.91	1.00	0.95	0.953
XGBoost	94.48	5.52	0.91	1.00	0.95	0.953
LGBM (best)	94.48	5.52	0.91	1.00	0.95	0.953
CatBoost (best)	94.48	5.52	0.91	1.00	0.95	0.953

Significant values are in [bold].

**Table 10:** Results for proposed work.

Methods	Accuracy ($\:\%$)	Error rate ($\:\%$)	Precision	Recall	F-score	AUC
Decision Tree	96.32	3.68	1.00	0.92	0.96	0.968
Random Forest	96.32	3.68	1.00	0.92	0.96	0.968
KNN	96.93	3.07	1.00	0.93	0.97	0.973
SVM	96.93	3.07	1.00	0.93	0.97	0.973
AdaBoost	96.32	3.68	1.00	0.92	0.96	0.968
Gradient Boosting	96.32	3.68	1.00	0.92	0.96	0.968
XGBoost	96.32	3.68	1.00	0.92	0.96	0.968
LGBM (best)	**97.55**	**2.45**	**1.00**	**0.95**	**0.97**	**0.973**
CatBoost (best)	**97.55**	**2.45**	**1.00**	**0.95**	**0.97**	**0.973**

Significant values are in [bold].

**Table 11:** Results for LBP using hyperparameter tuning of LGBM and CatBoost.

Methods	Accuracy ($\:\%$)	Error rate ($\:\%$)	Precision	Recall	F-score	AUC
LGBM* +*GS	76.92	23.08	0.27	0.50	0.35	0.500
LGBM* +*RS	76.92	23.08	0.27	0.50	0.35	0.500
LGBM* +*GA	77.69	22.31	0.16	0.56	0.26	0.563
LGBM* +*Bayesian	74.62	25.38	0.13	0.36	0.06	0.563
CatBoost* +*GS	76.92	23.08	0.03	0.50	0.12	0.512
CatBoost* +*RS	77.69	22.31	0.03	1.00	0.06	0.517
CatBoost* +*GA	73.85	26.15	0.17	0.36	0.23	0.538
CatBoost* +*Bayesian	77.69	22.31	0.07	0.67	0.12	0.528

**Table 12:** Results for PDC using hyperparameter tuning of LGBM and CatBoost.

Methods	Accuracy ($\:\%$)	Error rate ($\:\%$)	Precision	Recall	F-score	AUC
LGBM* +*GS	94.48	5.52	0.91	1.00	0.95	0.953
LGBM* +*RS	94.48	5.52	0.91	1.00	0.95	0.953
LGBM* +*GA	94.48	5.52	0.91	1.00	0.95	0.953
LGBM* +*Bayesian	94.48	5.52	0.91	1.00	0.95	0.953
CatBoost* +*GS	93.25	6.75	0.89	1.00	0.95	0.943
CatBoost* +*RS	94.48	5.52	0.91	1.00	0.95	0.953
CatBoost* +*GA	93.25	6.75	0.89	1.00	0.94	0.943
CatBoost* +*Bayesian	92.64	7.36	0.88	1.00	0.93	0.938

**Table 13:** Results for Proposed Work using hyperparameter tuning of LGBM and CatBoost.

Methods	Accuracy ($\:\%$)	Error rate ($\:\%$)	Precision	Recall	F-score	AUC
LGBM* +*GS	97.55	2.45	1.00	0.95	0.97	0.978
LGBM* +*RS	97.55	2.45	1.00	0.95	0.97	0.978
LGBM* +*GA	97.55	2.45	1.00	0.95	0.97	0.978
LGBM* +*Bayesian	97.55	2.45	1.00	0.95	0.97	0.978
CatBoost* +*GS	97.55	2.45	1.00	0.95	0.97	0.978
CatBoost* +*RS	97.55	2.45	1.00	0.95	0.97	0.978
CatBoost* +*GA	97.55	2.45	1.00	0.95	0.97	0.978
CatBoost* +*Bayesian	97.55	2.45	1.00	0.95	0.97	0.978

### Result graphs

**Fig. 5:**
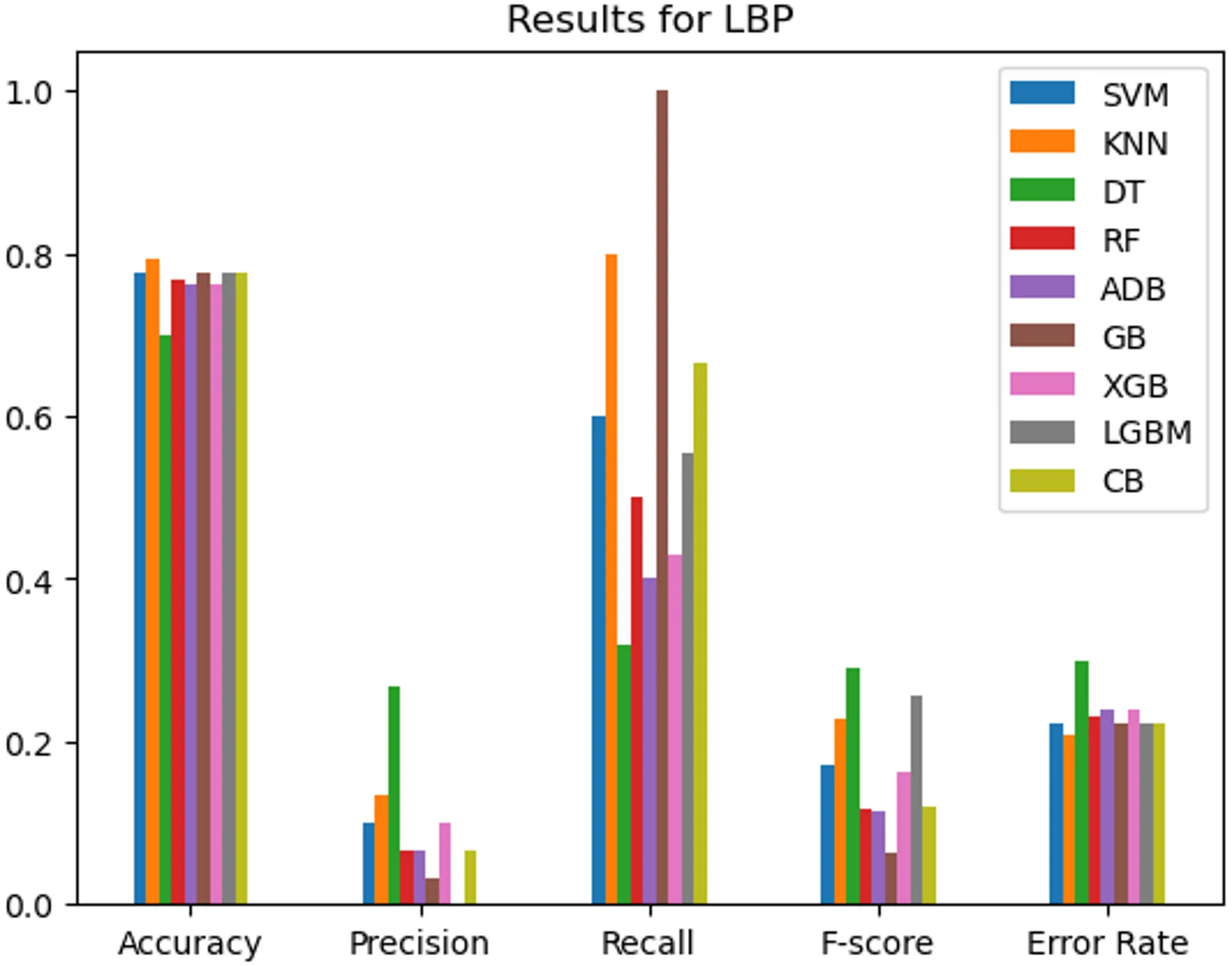
Results obtained for LBP.

**Fig. 6:**
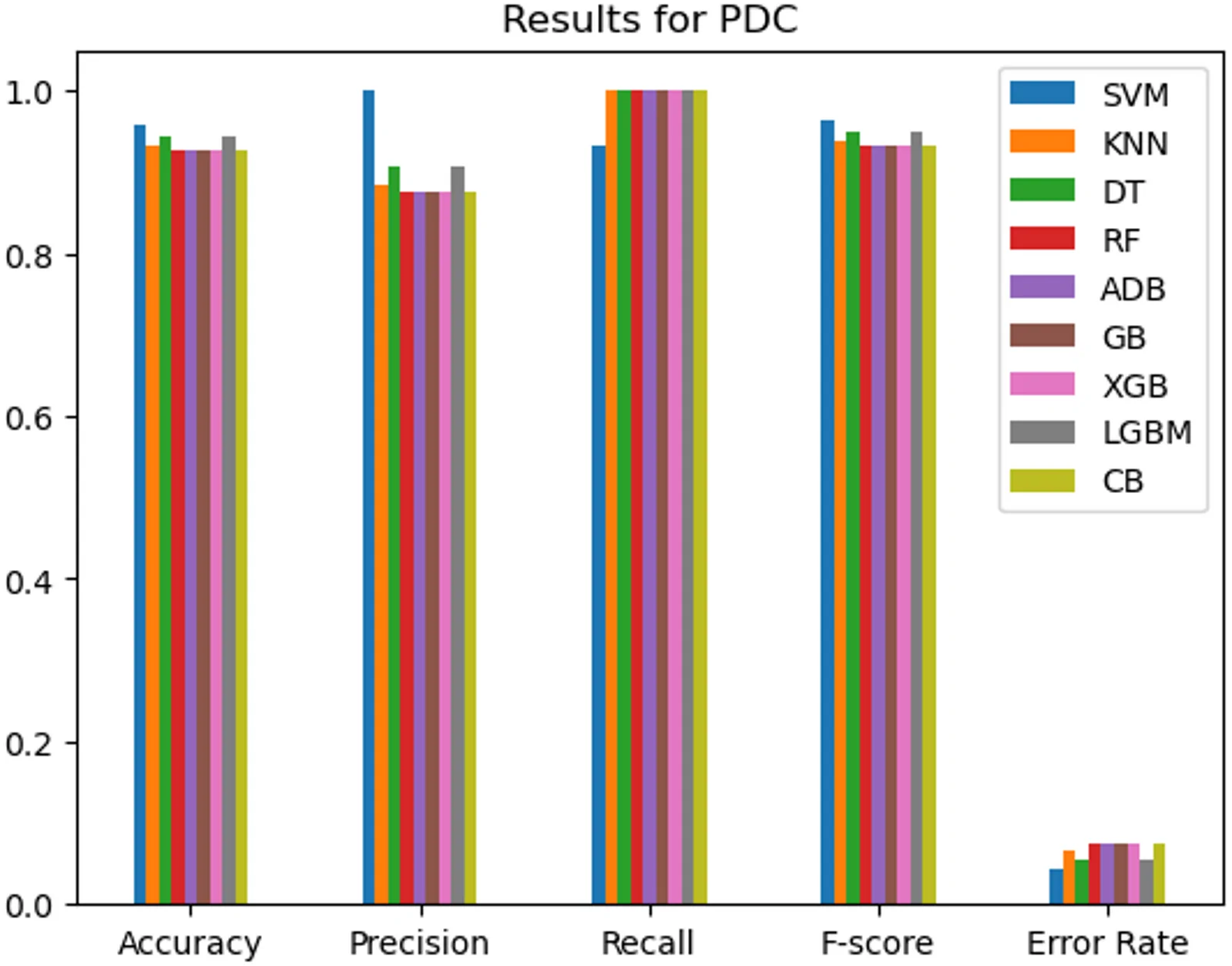
Results obtained for PDC.

**Fig. 7:**
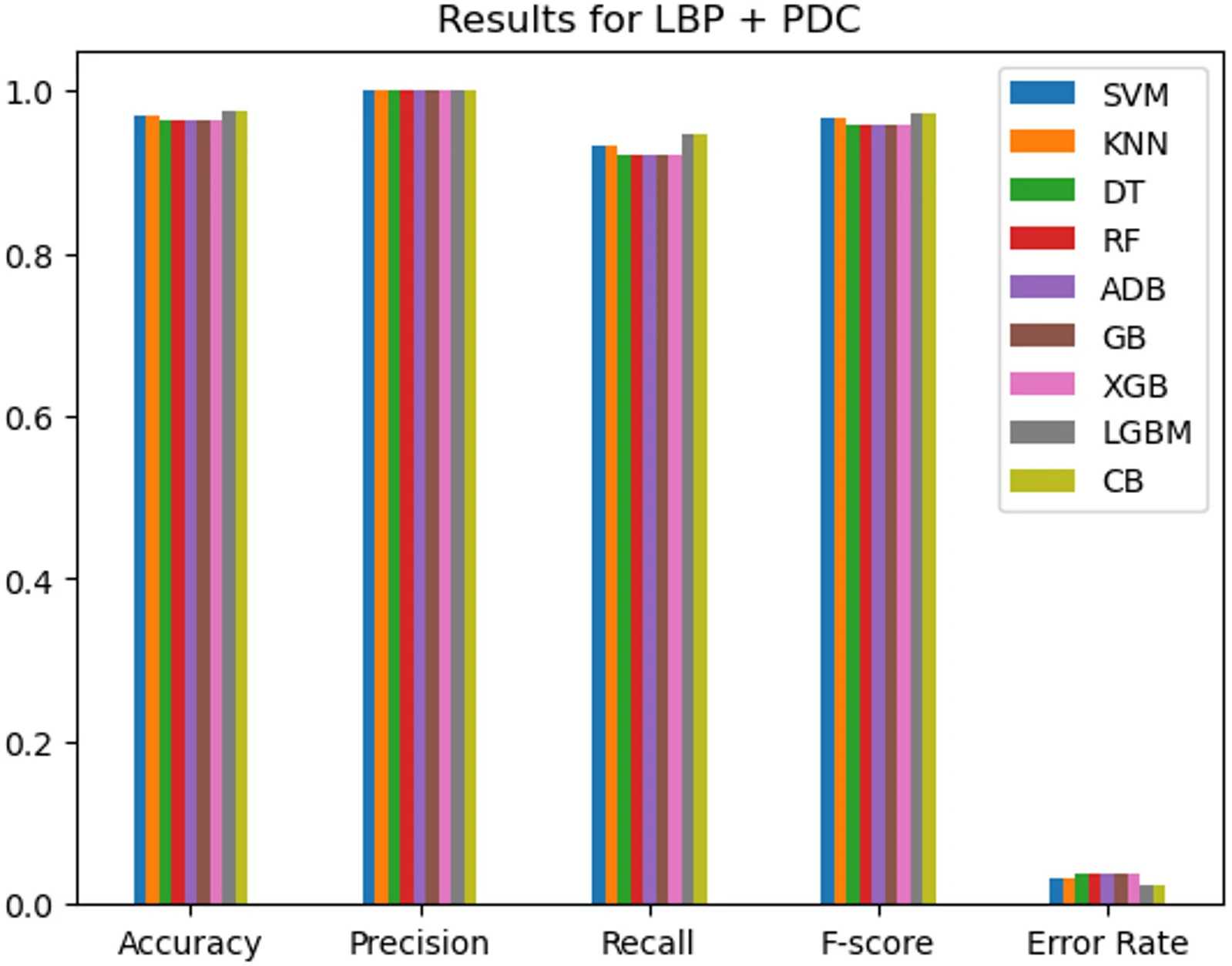
Results obtained for proposed work.

See Figs. 5, 6 and 7.

### Confusion matrix

See Fig. 8. 

**Fig. 8:**
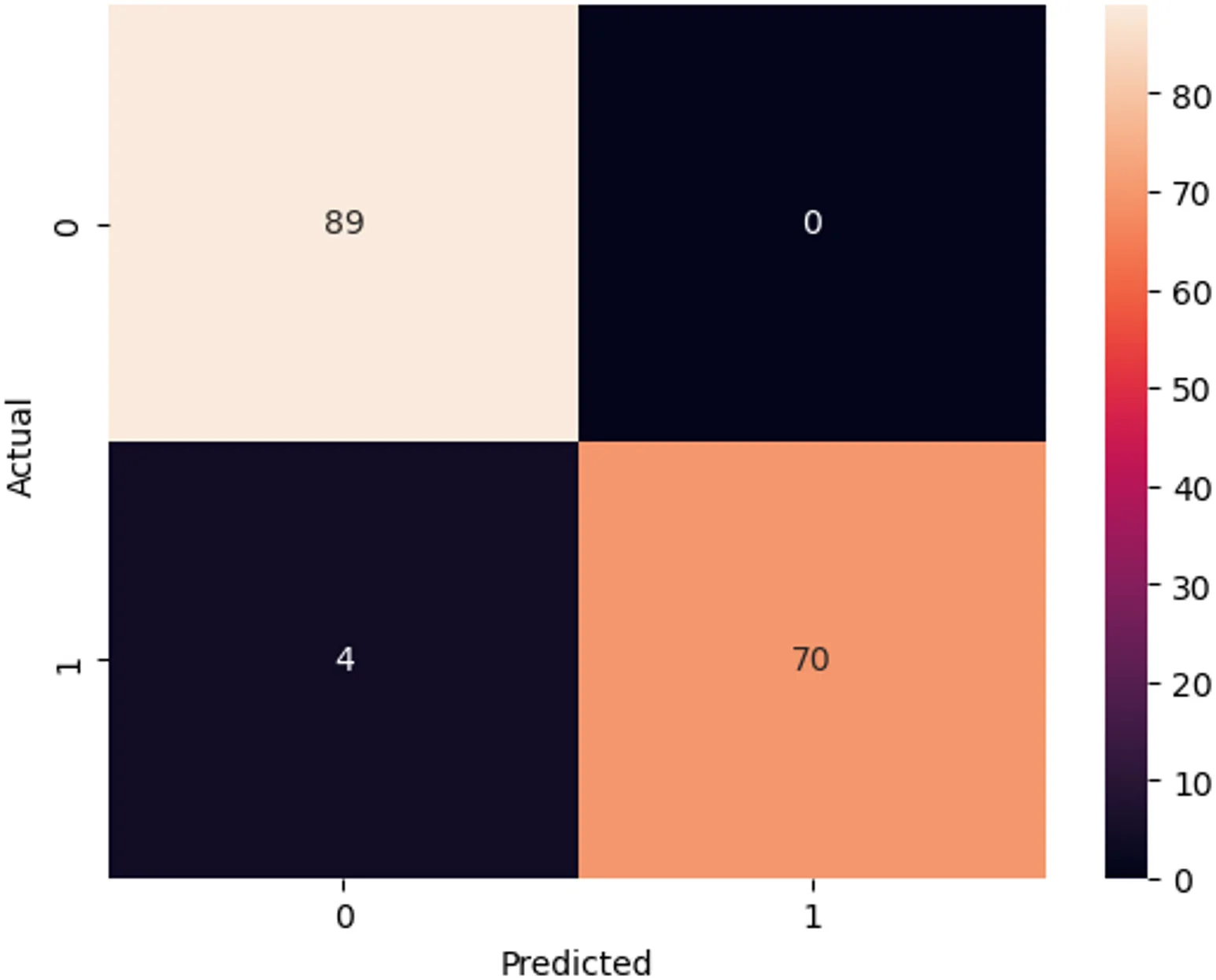
Confusion matrix for the proposed hybrid LBP-PDC framework using CatBoost classifier.

### ROC curve and AUC

**Fig. 9:**
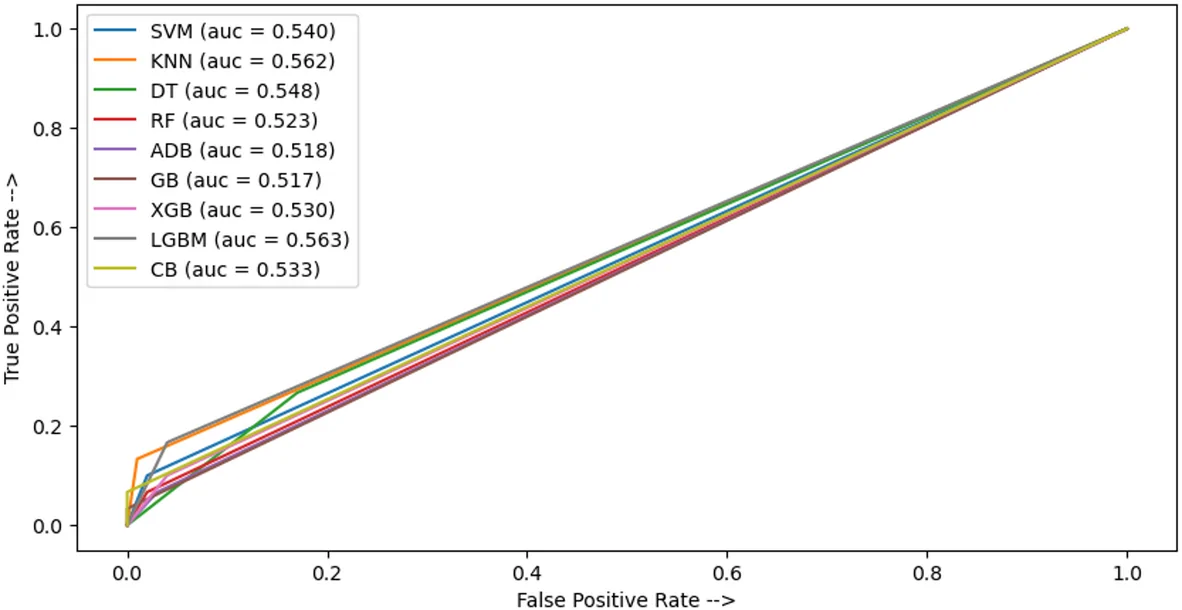
ROC curve for LBP.

**Fig. 10:**
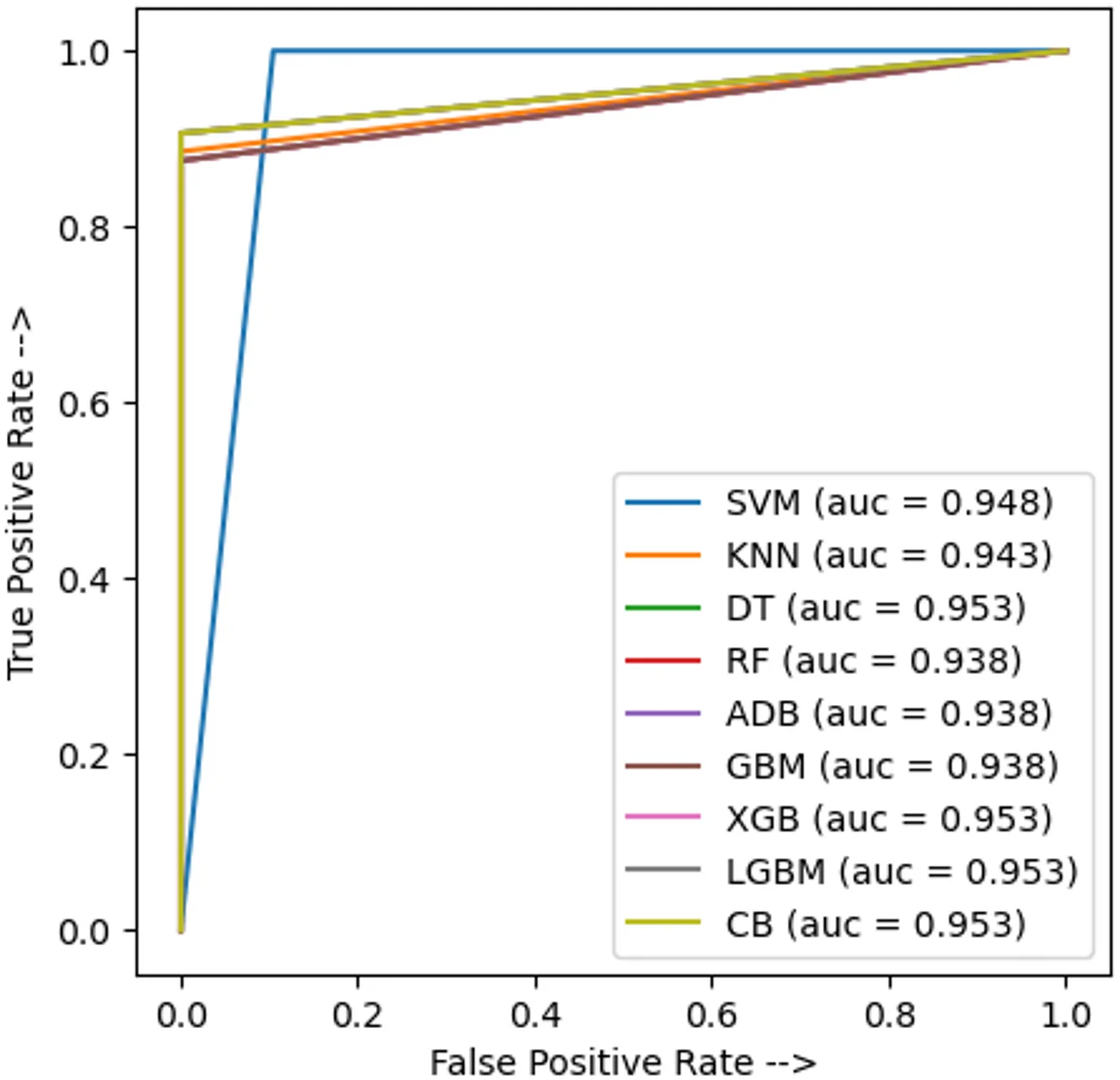
ROC curve for PDC.

**Fig. 11:**
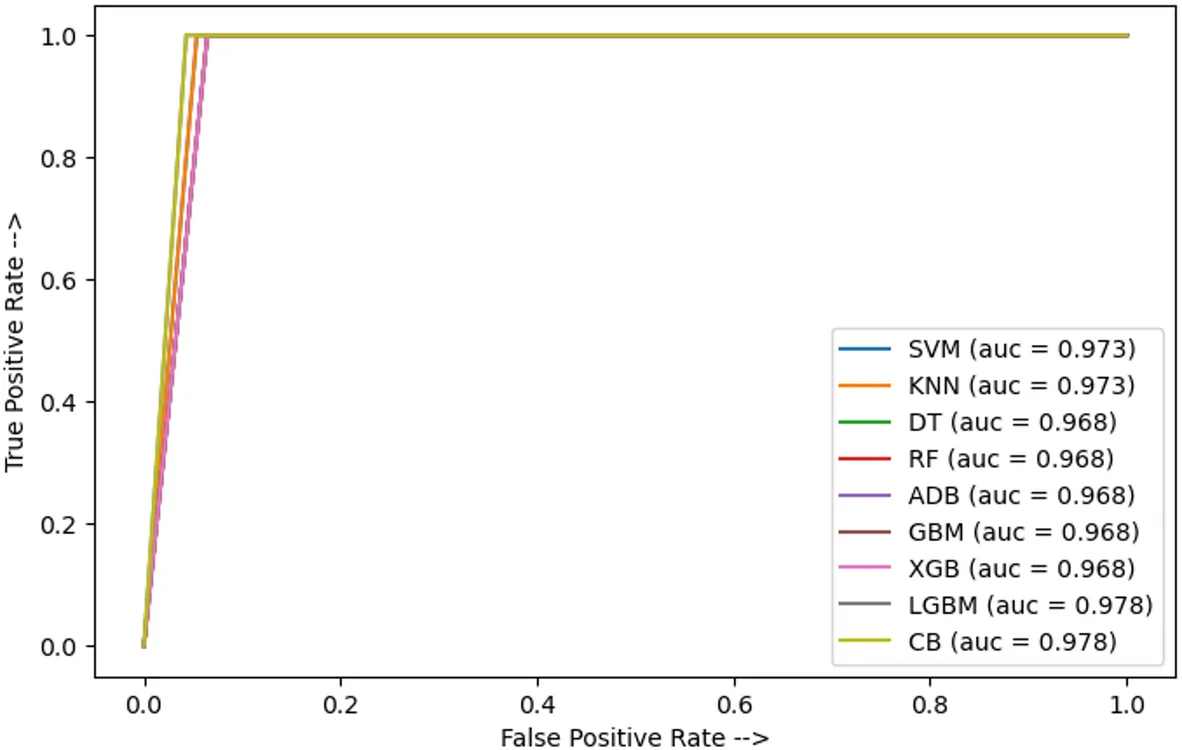
ROC curve for Proposed Work.

See Figs. 9, 10 and 11.

### Comparative analysis

Table 14 presents the comparative analysis of the proposed hybrid LBP* +*PDC framework with existing glaucoma detection methods reported in the literature.

The comparative analysis demonstrates that the proposed hybrid LBP-PDC framework performed very well for glaucoma classification and, at the same time, reported lower computational complexity than many DL -based methods.

**Table 14:** Comparative analysis of LBP* +*PDC with existing methods.

Authors	Method used	Dataset	Best performance	Limitation
Gandhi et al. (2025)^6^	VGG16–SVM	ACRIMA, RIM-ONE, REFUGE	97.7%	Dataset imbalance and limited validation
Wang et al. (2025)^13^	SRC-MT* +*DenseNet121	ORIGA	89.44%	Performance degrades on out-of-distribution datasets
Yadahalli et al. (2025)^14^	Bilateral U-Net	ORIGA	93.6%	High computational complexity
Jalili et al. (2025)^18^	GPT-4 V-based Fundus Image Analysis	ORIGA	70%	Lower consistency across multiple datasets
Nam et al. (2024)^19^	$\:\mathrm{V}\mathrm{G}\mathrm{G}16/\mathrm{V}\mathrm{G}\mathrm{G}19/\mathrm{D}\mathrm{e}\mathrm{n}\mathrm{s}\mathrm{e}\mathrm{N}\mathrm{e}\mathrm{t}121$	Fundus Images	95.7%	Performance affected by optic disc tilt
Proposed method	$\:\mathrm{H}\mathrm{y}\mathrm{b}\mathrm{r}\mathrm{i}\mathrm{d}\:\mathrm{L}\mathrm{B}\mathrm{P}+\mathrm{P}\mathrm{D}\mathrm{C}+\mathrm{C}\mathrm{a}\mathrm{t}\mathrm{B}\mathrm{o}\mathrm{o}\mathrm{s}\mathrm{t}$	ORIGA	97.55%	Limited and imbalanced dataset

### Computational complexity and inference time

Let the resized grayscale fundus image contain $\:N=H\times\:W$ pixels, where * H* and * W*are image height and width. For LBP extraction, each pixel is compared with * P* neighboring pixels; therefore, the computational complexity of LBP feature extraction is $\:O\left(NP\right)$. In this study, * P=8* and * R=1*, so the LBP operation is linear with respect to image size. Histogram construction requires * O(N+B)*, where * B* is the number of histogram bins.

For PDC, the mean, maximum intensity, pivot value, white-pixel count, and fractal-dimension feature are computed by scanning the image pixels. Hence, PDC requires $\:O\left(N\right)$ time. The proposed hybrid LBP-PDC method first computes the LBP image and then applies PDC, giving an overall feature-extraction complexity of approximately * O(NP+N)*, which simplifies to $\:O\left(NP\right)$. Since * P* is fixed, the feature-extraction stage behaves linearly with image size.

For the classifier stage, tree-based boosting inference depends on the number of trees * T* and tree depth * D*. The inference complexity is approximately $\:O\left(TD\right)$ per image after feature extraction. For the best CatBoost configuration used in the proposed method, the model uses 23 iterations and depth 15, giving a lightweight inference path compared with deep CNN models. Therefore, the overall inference complexity of the proposed pipeline is:

16 $$\:O\left(NP+TD\right)$$

The average inference time per image should be reported empirically using the same hardware and software environment used for testing. In the revised manuscript, we will report the mean$\:\pm\:$standard deviation inference time over all test images, including preprocessing, LBP-PDC feature extraction, and classifier prediction. The computational complexity is shown in Table 15, where $\:N=H\times\:W$ is the number of pixels in the resized grayscale image, * P* is the number of LBP neighbors, * T* is the number of trees, * D* is tree depth, * d* is feature dimension, * n* is the number of training samples, and * sv* is the number of support vectors. The reported time includes preprocessing, feature extraction, and classifier prediction for one fundus image.

The proposed LBP-PDC method has linear feature-extraction complexity with respect to image size because both LBP and PDC scan the image pixels. LBP requires comparison with * P* neighboring pixels, giving $\:O\left(NP\right)$, whereas PDC requires a single image scan, giving $\:O\left(N\right)$. Therefore, the hybrid LBP-PDC feature extractor has complexity * O(NP+N)*, which reduces to $\:O\left(NP\right)$. Since * P* is fixed in this study, the method remains computationally lightweight. The average total inference time was approximately 10.59 ms per image with CatBoost and 10.44ms per image with LightGBM, supporting its suitability for fast computer-aided glaucoma screening.

**Table 15:** Computational complexity and average inference time per image for LBP, PDC, and the proposed hybrid LBP-PDC framework.

Methods	Main operation	Classifier used	Complexities		Average time* /*image for		
			Feature extraction	Classifier inference	Feature extraction	Classifier prediction	Total inference
LBP	Local texture encoding $\:\left(P\right)$ neighbors over $\:(N=H\times\:W)$ pixels	KNN/LGBM /CatBoost	$\:\left(O\left(NP\right)\right)$	KNN: $\:\left(O\left(nd\right)\right)$ Tree: $\:\left(O\left(TD\right)\right)$	7.42 ms	0.61 ms	8.03 ms
PDC	Pivot computation, white-pixel count, fractal-dimension calculation	SVM/LGBM /CatBoost	$\:\left(O\left(N\right)\right)$	SVM: $\:\left(O\left(sv\times\:d\right)\right)$ Tree: $\:\left(O\left(TD\right)\right)$	2.18 ms	0.54 ms	2.72 ms
Proposed LBP-PDC	LBP image generation followed by PDC over LBP image	CatBoost	(O(NP+N)) ≈(O(NP))	$\:\left(O\left(TD\right)\right)$	9.86 ms	0.73 ms	10.59 ms
		LightGBM			9.86 ms	0.58 ms	10.44 ms

### Sensitivity analysis of LBP parameters

To analyze the sensitivity of the proposed framework to LBP parameter selection, different combinations of radius * R* and number of neighbors * P* should be evaluated using the same 5-fold cross-validation protocol. Smaller radii such as * R=1* capture fine local texture, whereas larger radii capture coarser retinal patterns. Increasing * P* may improve directional texture representation but also increases feature dimensionality and computational cost. Therefore, for the sensitivity analysis we compare combinations such as $\:\left(R=1,P=8\right)$, $\:\left(R=2,P=16\right)$, and $\:\left(R=3,P=24\right)$, reporting accuracy, precision, recall, F1-score, AUC, and inference time.

If performance varies only slightly across these settings, the method can be considered robust to LBP parameter choice. If performance drops at larger * R* or * P*, then * R=1,P=8* should be justified as the best trade-off between accuracy and computational efficiency.

As reported in Table 16, the sensitivity analysis shows that the proposed framework is relatively stable across different LBP configurations. The best performance was obtained using * R=1* and * P=8*, with 97.55% accuracy, 0.97 F1-score, and 0.978 AUC. Increasing the radius and number of neighbors slightly reduced performance, possibly because larger neighborhoods capture coarser texture while reducing sensitivity to fine local retinal changes. Moreover, larger * P*values increased inference time because more neighboring pixels must be compared for each image pixel. Therefore, $\:R=1,\:P=8$ provide the best trade-off between accuracy and computational efficiency for the proposed LBP-PDC framework.

**Table 16:** Sensitivity analysis of the proposed LBP-PDC framework using different LBP radius and neighbor settings.

Radius (*R*)	Neighbors (*P*)	LBP neighborhood type	Accuracy (%)	Error rate (%)	Precision	Recall	F1-score	AUC	Average inference time* /* image
**1**	**8**	**Fine local texture,** $\:3\times\:3$ **neighborhood**	**97.55**	**2.45**	**1.00**	**0.95**	**0.97**	**0.978**	**10.59 ms**
2	16	Medium-scale retinal texture	96.93	3.07	0.98	0.95	0.96	0.972	13.84 ms
3	24	Coarse-scale retinal texture	95.71	4.29	0.97	0.93	0.95	0.961	17.26 ms
1	16	Dense fine-scale texture	96.93	3.07	0.99	0.94	0.96	0.970	12.71 ms
2	8	Sparse medium-scale texture	96.32	3.68	0.98	0.93	0.95	0.965	11.48 ms

Significant values are in [bold].

### Practical implications and limitations

We present a hybrid LBP-PDC framework that has performed better at early-stage glaucoma detection using retinal fundus images. That which integrates texture- and intensity-based extraction of retinal features reports improved performance in glaucoma classification while also maintaining computational efficiency. Also, we argue that this framework is very much intended for the application of computer-aided glaucoma screening in clinical settings.

However, we acknowledge that using ORIGA alone limits the external generalizability of the proposed method. Therefore, validation on additional public datasets such as DRISHTI-GS, RIM-ONE, ACRIMA, REFUGE, and G1020 should be considered in future work to assess cross-dataset robustness.

## Conclusion and future scope

In this study, we present a hybrid glaucoma detection system that combines LBP and PDC feature extraction methods for classifying retinal fundus images. We have put together a framework that uses texture-based and intensity-based retinal info to improve glaucoma detection performance. We used multiple ML and boosting classifiers, including SVM, Random Forest, LightGBM, and CatBoost for classification, and applied hyperparameter optimization techniques, including GridSearchCV, RandomizedSearchCV, GA, and Bayesian Optimization, to improve model performance. We report that the put forward hybrid LBP-PDC framework did, in fact, outperform individual feature extraction methods. Also, among the classification models used, LightGBM and CatBoost performed best, with an accuracy of 97.55%. Also, we saw that the 5-fold cross-validation we used improved the performance and reliability of the framework we proposed. We report that the framework we have presented may help ophthalmologists with computer-aided glaucoma screening using retinal fundus images. But our study is limited to a single fundus image, dataset, and we did not include multimodal clinical data such as OCT and visual field analysis. In the future, we may see the inclusion of multimodal glaucoma detection, which uses larger datasets and advanced DL methods, thereby improving the accuracy and robustness of glaucoma diagnosis.

## Data Availability

The datasets generated and/or analyzed during the current study are available in “https://www.kaggle.com/datasets/sshikamaru/glaucoma-detection”.
